# Intravenous Rehydration for Severe Acute Malnutrition with Gastroenteritis

**DOI:** 10.1056/NEJMoa2505752

**Published:** 2025-06-13

**Authors:** Kathryn Maitland, San Maurice Ouattara, Hadiza Sainna, Abdullahi Chara, Oluwakemi F. Ogundipe, Temmy Sunyoto, M. Hamaluba, Peter Olupot-Olupot, Florence Alaroker, Roisin Connon, Amadou Saidou Maguina, William Okiror, Denis Aromut, Eric Mwajombo, Emmanuel Oguda, Christabel Mogaka, Céline Langendorf, Juan Emmanuel Dewez, Iza Ciglenecki, Diana M. Gibb, Matthew E. Coldiron, Roberta Petrucci, Elizabeth C. George, for the GASTROSAM trial group

**Affiliations:** Institute of Global Health and Innovation, Department of Surgery and Cancer Paediatrics; Kenya Medical Research Institute (KEMRI)–Wellcome Trust Research Programme, Kilifi, Kenya; Imperial College, London UK; Epicentre, Magaria, Niger; Médecins Sans Frontières, Maiduguri, Nigeria; Médecins Sans Frontières, Maiduguri, Nigeria; Médecins Sans Frontières Brussels, Belgium; Soroti Regional Referral Hospital, Soroti; Médecins Sans Frontières Brussels, Belgium; Kenya Medical Research Institute (KEMRI)–Wellcome Trust Research Programme, Kilifi, Kenya; Busitema University Faculty of Health Sciences, Mbale Regional Referral Hospital Mbale; Medical Research Council Clinical Trials Unit at University College London; Médecins Sans Frontières, Magaria, Niger; Busitema University Faculty of Health Sciences, Mbale Regional Referral Hospital Mbale; Soroti Regional Referral Hospital, Soroti; Kenya Medical Research Institute (KEMRI)–Wellcome Trust Research Programme, Kilifi, Kenya; Kenya Medical Research Institute (KEMRI)–Wellcome Trust Research Programme, Kilifi, Kenya; Kenya Medical Research Institute (KEMRI)–Wellcome Trust Research Programme, Kilifi, Kenya; both in Uganda. Epicentre, Paris, France; London School of Hygiene and Tropical Medicine, London UK; Médecins Sans Frontières, Geneva, Switzerland; Médecins Sans Frontières, Geneva, Switzerland; Medical Research Council Clinical Trials Unit at University College London; both in Uganda. Epicentre, Paris, France; Médecins Sans Frontières, Geneva, Switzerland; Medical Research Council Clinical Trials Unit at University College London

## Abstract

**Background:**

International recommendations advise against giving intravenous rehydration to children with severe acute malnutrition (SAM), given concerns about fluid overload; but supportive evidence is lacking. High mortality rates suggest adopting intravenous rehydration strategies might improve outcomes.

**Methods:**

This study, the GASTROSAM factorial open-label superiority trial, performed in four African countries, randomized children aged 0.5-12 years with SAM and dehydrating diarrhea (2:1:1) to control (oral rehydration only, plus intravenous boluses for shock); to liberal intravenous management-- rapid rehydration (100ml/kg Ringer’s Lactate) over 3-6h, with boluses for shock) (liberal:rapid); or to slow rehydration (100ml/kg Ringers Lactate over 8 hours, no boluses) (liberal:slow). A second randomization compared two oral rehydration solutions (ORS) (reported elsewhere). The primary endpoint was 96-hour mortality.

**Results:**

Two-hundred-seventy-two children (median age 13months) were randomized to control (n=138); liberal:rapid(n=67) or liberal:slow(n=67) rehydration and followed for 28 days (loss-to-follow-up, 4(1%)). Nasogastric tubes for ORS were required in 126(93%) control versus 82(65%) of pooled liberal intravenous arms. Twelve control participants (9%) received intravenous boluses at admission versus 7 liberal:rapid (10%) and no liberal:slow participants. Eleven controls (8%) compared with 9 liberal rehydration participants (7%) [5 rapid and 4 slow] died before 96hours (liberal vs. control, Risk Ratio=1.02(9 CI 0.38-2.39);p=0.69). Seventeen control (12%) compared with 14 liberal group participants (10%) died before 28 days (liberal vs. control:Hazard Ratio(HR)=0.85(0.41-1.76)). Serious adverse events occurred in 32 control (23%), 14 liberal:rapid (21%), and 10 liberal:slow group participants (15%). No evidence of pulmonary edema, heart failure or fluid overload was noted.

**Conclusions:**

There were no evident differences in mortality at 96 hours.

(Funded by the Joint Global Health Trials Scheme of the United Kingdom’s Medical Research Council and others; GASTROSAM Current Controlled Trials number, ISRCTN76149273)

**I**n 2016 the World Health Organization (WHO), United Nations Children’s Fund and World Bank Group Interagency estimated that 17 million children under five years were severely undernourished^1^. The most extreme form, severe acute malnutrition (SAM), is a leading cause of pediatric hospital admissions in Africa^2,3^. Many such children have additional complications including marked dehydration due to diarrhea,^4^ which is associated with high in-hospital mortality(27-41%)^4–6^. Current recommendations for rehydration in children with SAM differ from those without SAM^7^. First, intravenous rehydration is not recommended in SAM on the grounds that malnourished children are at high risk of cardiac compromise and sodium overload^8,9^. Second, low sodium oral rehydration solutions (ORS) are not recommended for similar rationale. Although such guidance documents have been in place for over two decades, no prior^10^ or subsequent evidence has been provided to support these recommendations^11,12^. Thus, oral rehydration is the recommended option with intravenous boluses provided only in the event of shock. This approach results in children requiring a nasogastric tube to administer oral rehydration, since most are unable to take or retain oral fluids. Additionally, most cases are managed on busy, overcrowded pediatric wards or dedicated nutrition units with limited numbers of nursing staff who can ensure close supervision to support safe implementation of oral-nasogastric rehydration and monitor for signs of shock. Shock complicates ~25% of pediatric cases with severe dehydration^13^ with high in-hospital mortality (>40%)^13–15^.

Physiological studies have supported the safety of intravenous fluids in severe malnutrition, with no evidence of cardiac compromise^16,17^. A cohort study that matched hospitalized SAM cases by co-morbidity to non-malnourished controls, also found no evidence that children with SAM were more likely than others to have cardiac dysfunction or arrhythmias^16^. Fluid-responsiveness was demonstrated on the Frank-Starling curves in cases receiving rehydration or boluses^15^. Taking the results of such studies together, randomized controlled trials (RCT) seem indicated^11^.

The intravenous rehydration strategy recommended by WHO for non-malnourished children with gastroenteritis and severe dehydration (estimated 10% loss,~100ml/kg) is called ‘Plan C’^7^. It includes two phases of intravenous rehydration (an initial fast component followed by a slower phase over 4-6 hours) with rates differing for infants and children over one year, plus fluid boluses (20ml/kg) for hypovolemic shock. Since the Fluid Expansion As a Supportive Therapy (FEAST) trial demonstrated harm from fluids boluses in African children with non-hypovolemic shock^18^ we were concerned that such harm might extend to aggressive rehydration for severe dehydration. In a RCT enrolling non-malnourished children^19^ with severe dehydration due to gastroenteritis in Uganda and Kenya, we compared Plan C (liberal:rapid) to an equivalent volume of intravenous rehydration (100ml/kg) given slowly over 8 hours (without boluses for shock). The slow strategy had outcomes similar to those of Plan C and was simpler to implement, requiring less oversight^20^.

Here we hypothesized that liberal intravenous rehydration (either given rapidly or slowly) compared with standard oral rehydration would reduce mortality in children hospitalized with SAM complicated by gastroenteritis^21^.

## Methods

### Trial Design and Oversight

We conducted an investigator-initiated open-label, superiority, multicenter, factorial randomized trial in six hospitals in Uganda (n=2), Kenya (n=2), Niger (n=1) and Nigeria(n=1), detailed in Supplementary Methods. Of note, participants from Niger and Nigeria comprise more than 90% of the cohort. The protocol^21^ was approved by the local ethics committees and is available at nejm.org. KM, DMG, POO and ECG designed the study; SO,HS,AC,AS,WO,DA,EM,EO gathered the data; OFO,TS,MH,POO,FA,CL,JED,IC,MEC and RP supervised the study teams. The first author wrote the first draft of the manuscript, which was reviewed and agreed on by all the authors. CM, RC and ECG vouch for the for the accuracy and completeness of the data.

### Trial Population

Eligible children aged 6 months to 12 years hospitalized with SAM (defined as either weight-for-height z-score <-3, mid-upper arm circumference (MUAC) <11.5cm or presentation with edematous malnutrition (kwashiorkor) with at least bilateral pedal edema^7^) with gastroenteritis (>3 loose stools per day) and signs of severe dehydration were included. Severe dehydration signs, following WHO criteria, include two or more of the following-- altered consciousness based on a score of <15 on the four-component AVPU score of 3-15, where Alert is 15, responsiveness to Verbal or Painful stimuli are next and Unresponsive is the final category, sunken eyes, reduced skin turgor (slow abdominal skin pinch return>2s) or unable to take or retain oral fluids. Children with known congenital or rheumatic heart disease or with non-acute diarrhea (lasting>14 days) were excluded.

### Screening and Randomization

All children with SAM admitted to hospital with an acute history of gastroenteritis were screened for inclusion by study staff. In Niger and Nigeria participants were transferred to an intensive care ward (although assisted ventilation was not available) for management by a dedicated study team (see Supplementary Appendix, Table S1 for generalizability). In Uganda and Kenya participants were managed on general pediatric wards. When prior written consent from parents or legal guardians could not be obtained, ethics committees approved verbal assent with delayed written informed consent as soon as practical^22^. Otherwise, written informed consent was obtained from parents or guardians before randomization. The statistician in London generated the sequential randomization list, computer-generated using variably-sized permuted blocks.

Randomization at sites used consecutively numbered opaque sealed envelopes containing the randomized allocation, opened in numerical order.

Participants were randomly assigned 2:1:1 to control (WHO SAM strategy): oral rehydration solution 5ml/kg every 30 minutes for the first 2hours followed by 5–10 ml/kg per hour for the next 4–10h alternating hourly with F75 milk formula, with boluses of Ringer’s Lactate (15 ml/kg) for those with shock); liberal:rapid rehydration (WHO Plan C: 100 ml/kg Ringer’s Lactate over 3-6 hours according to age with boluses (20 ml/kg) for those with shock) or liberal:slow rehydration (100 ml/kg Ringer’s Lactate over 8h and no boluses) (Tables S2-S5; Fig. S1). Shock was defined as all of the following: cold peripheries (meaning hands and-or feet)_, a weak and fast pulse (rate not specified) and a capillary refilling time >3s^15^.

For oral rehydration management, all participants were simultaneously factorially randomized (1:1) either to (Rehydration Solution for Malnutrition (ReSoMal)) or WHO oral rehydration solution (ORS) (recommended for non-SAM) (comparison reported separately, Supplementary Methods).

### Study Procedures and Follow-up

Basic infrastructural support for emergency care, patient monitors, bedside hemoglobin, glucose and lactate point-of-care tests were provided. Bedside observations were performed at admission and every 30 minutes for the first 2 hours, hourly to 8hours, then at 12, 24, 36 and 48hours after randomization. Clinical chemistry was assessed at 0, 8 and 24hours. Blood cultures were performed where facilities permitted. Participants unable to tolerate oral fluids had a nasogastric tube placed to administer oral rehydration fluids and nutritional milk (called F75); its correct positioning was checked at each administration. Participants were actively monitored for serious adverse events (SAEs), particularly suspected cardiac or pulmonary overload, at each clinical assessment. Participants were clinically assessed at 7-days and 28-days (trial exit) post-randomization. Study staff were unblinded throughout; laboratory tests were assayed blinded.

### End Points

The primary endpoint was mortality at 96hours. Secondary efficacy endpoints were mortality to day-28; change in weight, MUAC, at Day 3 and Day 7; urine output at 8hours. Safety endpoints were evidence of pulmonary edema or heart failure; change in sodium at 24hours compared with 8hours (post intravenous strategy completion); and correction of electrolyte abnormalities (severe hyponatremia <125 mmol/L or hypokalemia <2.5mol/L).

### Statistical Analysis

Enrolling 272 children with severe dehydration was anticipated to provide 80% power to detect a 30% relative reduction in 96-hour mortality from 58% in the control group to 41% in the liberal strategies, assuming no lost-to-follow-up by 96hours (2-sided alpha=0.05) (Supplementary Appendix, Methods). An independent Data Monitoring Committee reviewed interim data (four meetings). This report presents the pre-specified primary comparison for the liberal intravenous rehydration randomizations (pooling liberal:rapid and liberal:slow intravenous rehydration arms) vs. control, and also analysing separately vs. control. Randomized groups were compared with an intention-to-treat analysis using a Mantel-Haenszel adjusted risk ratio for mortality at 96hours (primary endpoint), adjusted for pre-specified covariate of site (hospital), and Cox regression for mortality by 28 days (secondary endpoint). Continuous outcomes were compared using linear regression to estimate mean difference and confidence intervals at each time point, and proportions using chi-squared tests (prespecified in the Statistical Analysis Plan (SAP): presented as odds ratios and confidence intervals from logistic regression). Time to correction of hyponatremia and hypokalemia were calculated using competing risks regression taking into account death. Confidence intervals were not adjusted for multiplicity and may not be used in place of hypothesis testing. Complete case analyses are presented for primary and secondary outcomes following the SAP under a missing completely at random assumption as missingness was evenly distributed between arms and below the pre-defined threshold (10%); however, for secondary outcomes, where missingness was close to the threshold, multiple imputation was conducted under the missing at random assumption (Supplementary Appendix, Methods). Analyses used Stata v18.

## Results

**Figure 1:**
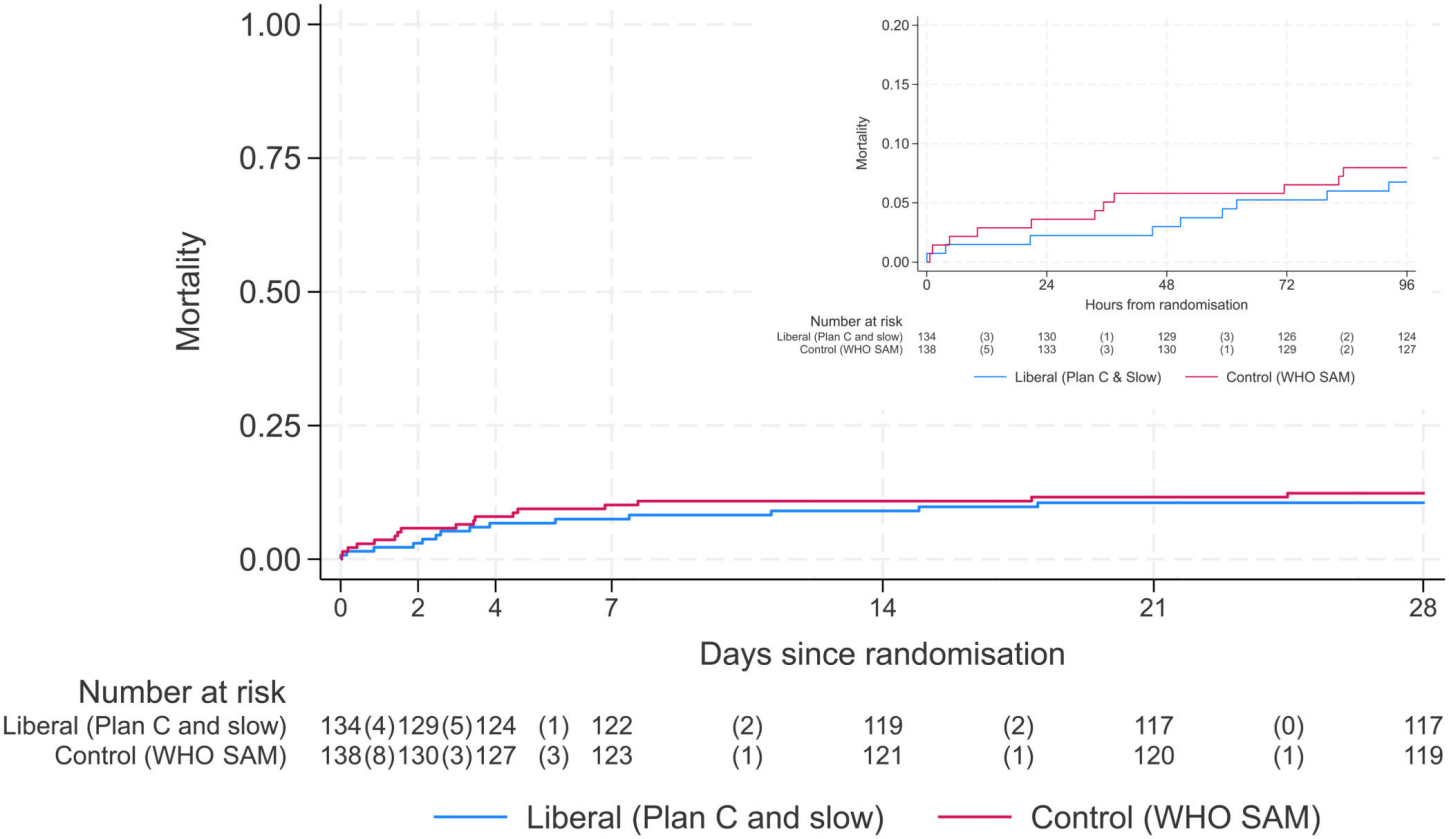
Mortality at 96 Hours and 28 Days Depicted here are mortality at 96 Hours (insert) and 28 days. The figure compares control with pooled liberal.

Between September 2nd 2019 and October 27^th^ 2024, 272 participants were randomized--138 to control and 134 to liberal intravenous rehydration (67 liberal:rapid, 67 liberal:slow); all are included in all analyses (Fig.1;Fig.S2). Recruitment was halted between March 2020 through November 2021 due to COVID (Fig.S3). There were few imbalances in baseline characteristics between randomized groups (Table 1), fewer than expected by chance. Most children had three or more signs of dehydration (267(98%) sunken eyes, 242(89%) decreased skin turgor); 215(79%) were unable to take or retain oral fluids) and 76(29%) had moderate hypotension. Previously identified risk factors for mortality^4,5^ were highly prevalent including impaired consciousness (104:38%), bacteremia (largely gram-negative) (12/98 tested:12%); severe hyponatremia (sodium <125mmol/L) (137(52%)) and hypokalemia (potassium <2.5 mmol/L) (115(45%)). Few had kwashiorkor (11(4%)) or known HIV (2(1%)).

### Adherence to Randomized Strategy and Clinical Management

Thirty-one (22%) participants in the control arm received intravenous fluids within 24h, starting median(IQR) 123(13-470) mins from randomization; including 12(9%) with shock receiving immediate boluses and 14(9%) following later development of shock or another serious adverse event (Table 2; Table S6). Sixty-six children (99%) in the liberal:rapid arm received intravenous fluids starting median(IQR) 16(10-28) mins from randomization; 7 of 8 with shock received an immediate bolus (1 died prior to bolus administration). Sixty-seven participants (100%) in the liberal:slow arm received intravenous fluids without boluses (5 with shock at baseline) starting median(IQR) 12(8-22) minutes from randomization). Oral rehydration started in 135/138(98%) participants in the control arm (2 died before starting; 1 missing form) (median(IQR) 0.3(0.2-0.5) hours), with 126 (92%) requiring a nasogastric tube. Post-intravenous rehydration ORS started in 64 children in the liberal:rapid arm (1 died before starting; 1 missing form) (median(IQR) 5.3(4.0-7.0) hours) (67% via nasogastric tube), and 62 in the liberal:slow arm (2 missing forms) (median 8.7 (8.4-9.2) hours) (63% via nasogastric tube). Vomiting and nasogastric tube insertion to administer oral rehydration were greater in the control group (Table 2).

### Mortality

At 96hours (primary endpoint) and day-28 (end of follow-up), 271 (99%) and 267 (98%) children, respectively, had known vital status. By 96hours, 11 (8%) in control arm vs. 9 (7%) in liberal rehydration (5 (7%) liberal:rapid; 4 (6%) liberal:slow) had died (Adjusted Risk Ratio (aRR) (liberal vs. control) =1.02 (95% CI 0.41,2.52); p=0.69, Table 3;Fig.1). At day-28 17(12%) control vs. 14(10%) liberal rehydration (8(12%) liberal:rapid, 6(9%) liberal:slow) participants had died (Hazard Ratio (HR)=0.85 (0.41,1.78)). Separate comparisons of 96-hour mortality were for liberal:rapid vs. control aRR=1.16 (0.40,3.40) and liberal:slow vs. control aRR=0.89 (0.28,2.80) (Tables S7-S8; Figs. S4-S8). Findings for the primary outcome were consistent across four prespecified subgroups: oral rehydration solution randomization, age (< or ≥1y), consciousness level and respiratory distress at randomization (Table S9).

### Safety and Other Endpoints

No child developed pulmonary edema or signs consistent with heart failure in the trial. There was no evidence of a difference between arms for Serious Adverse Events (SAEs; Table 3 and Tables S10, S11). Deterioration in consciousness level or seizures occurred in 18(13%) control and 10(8%) liberal rehydration (odds ratio (OR) liberal vs. control=0.54 (95%CI 0.24-1.23)). Shock developed in 11(9%) vs. 6(5%) respectively (odds ratio liberal vs. control OR=0.55 (95%CI 0.19-1.53). By 8h and 24h significantly more children randomized to control had severe hyponatremia (58/126(45%) and 35/126(27%) respectively) than liberal rehydration (20/128(16%) and 21/127(17%) respectively) (liberal vs. control OR=0.23(95%CI 0.13-0.41) and OR=0.53 (95%CI 0.29-0.98) respectively) (Table 3). The liberal arms corrected their severe hyponatremia quicker (sub-HR=1.55 (1.14,2.09)). Potassium increased more slowly with liberal arms at 8h and 24h (liberal vs. control mean (95%CI) difference -0.3 (-0.5,-0.2) and -0.4 (-0.6,-0.2) respectively) but there was no evidence of difference in time to correction of severe hypokalemia (sub-HR=0.87 (0. 57,1.19)). Day-3 weight increased more with liberal rehydration (+0.5 (0.4,0.5) vs. +0.4 (0.3,0.4) kg, mean difference +0.1 (0.1,0.2)) but there was no evidence of difference by day-7 (Table 3). Similar findings were reflected in other anthropometric measures (Tables S7-8). Results from complete case analyses were not sensitive to the missing completely at random assumption (Table S12).

## Discussion

This multicenter trial conducted in resource-poor conditions did not observe a reduction in mortality between standard and liberal rehydration strategies in patients with SAM. The use of liberal rehydration strategies, both intravenous and oral were not associated with cardiac or pulmonary complications and resulted in fewer patients requiring fluid boluses for shock and placement of nasogastric tubes.

The key limitation of our trial was the much lower overall mortality (9%) than we predicted from two small studies, which reported mortality at hospital discharge or 28 days as 68-82%^14,15^, at the high end compared with other observational data. A key reason for low mortality in our trial may be that most children were managed on high dependency wards (or step-down, intermediate care units) by dedicated clinical trial teams with very close and frequent monitoring to identify and treat complications (specifically fluid overload, shock or hypoglycemia) and to ensure protocol adherence. These measures were put in place to address concerns by ethics committees over the balance of risk to benefit for children participating in the trial, but may reduce the generalisability of the findings. The concerns resulted from longstanding national and international guidance recommending against IV rehydration in children with SAM arising from the perceived risk of incipient heart failure^7^. In routine practice in low-resourced, overcrowded pediatric wards in Africa, the close clinical monitoring afforded by our trial is not possible, as evidenced by the poor outcomes reported in SAM with severe dehydration under routine surveillance^4–6^. This underscores the need for simplified protocols for the management of dehydration. For example, at admission 79% of children were unable to take oral rehydration, resulting in 93% of the control arm requiring nasogastric tubes for oral rehydration solution administration. This is not a trivial low-risk procedure, especially in children with impaired consciousness and high purging rates. The current recommendations have resulted in additional demands on limited ward personnel, since oral rehydration solution cannot be given by the child’s caregiver (as noted in guidance documents). In contrast, slow intravenous rehydration, even compared to rapid rehydration, was simpler to implement requiring no calculation of volumes for boluses and adjustment for the rapid and slower rehydration phases depending on age.

Another limitation was low participant numbers with kwashiorkor, a key group expected to be at significant risk of heart failure. However, our research group has previously demonstrated that myocardial function is preserved in children with SAM with no difference in fractional shortening (a global measure of myocardial function) in children with marasmus (severe wasting) and kwashiorkor^16^. Thus, results are likely to be generalizable to this subgroup.

Relevant to the broader population of children hospitalized with acute diarrhea with severe dehydration (~10% weight loss), a study showed that approximately 20% temporarily fulfilled anthropometric criteria for SAM (MUAC<11.5cm) but were ‘reclassified’ as undernourished following rehydration.^24^ Thus, through ‘slippage’ the current recommendations may have wider implications, as potentially 20% of non-SAM children could be inappropriately diagnosed as malnourished and rehydrated. This may have contributed to the poor outcomes observed in the Global Enteric Multicentre (GEMS) study^23^.

Currently, at the bedside, clinicians need to consider nutritional status, age and the presence of shock to determine which rehydration strategy to follow. Given the findings of this study, in the absence of other data, we would suggest that current guidance be reviewed to consider simplifying rehydration protocols, removing the distinction in management between SAM and non-malnourished children, which would be more pragmatic in the under-resourced settings where most children are managed.

While there was no evidence of a difference in mortality at 96 hours between the rehydration strategies evaluated in our trial, power to detect modest differences was low. Specifically, there was no apparent signal of harm for the liberal intravenous rehydration strategies including no evidence of fluid overload nor evidence of sodium overload compared with the currently WHO-recommended oral rehydration strategy.

In summary, we detected no difference in mortality among the rehydration strategies used in children in the present factorial open-label superiority trial.

Disclosure forms provided by the authors are available with the full text of this article at NEJM.org.

## Supplementary Material

Supplement

## Figures and Tables

**Table 1:** Characteristics of Children at Baseline

	Control (N=138)	Liberal (Liberal: rapid and Liberal:slow combined) (N=134)	Liberal:rapid intravenous rehydration (N=67)	Liberal:slow intravenous rehydration (N=67)
Sex - male	62 (45%)	64 (48%)	28 (42%)	36 (54%)
Age months	12 (9, 22)	14 (9, 23)	16 (9, 24)	14 (9, 22)
Weight (kg)	5.3 (4.5, 6.1)	5.2 (4.6, 6.1)	5.0 (4.5, 6.1)	5.3 (4.7, 6.1)
Weight-for-height z-score	-4.7 (-5.5, -4.2)	-4.9 (-5.8, -4.2)	-5.0 (-5.8, -4.3)	-4.9 (-5.7, -4.2)
MUAC (cm)	10.5 (9.6, 11.0) [N=137]	10.1 (9.5, 11.0) [N=134]	10.0 (9.5, 10.8) [N=67]	10.4 (9.6, 11.0) [N=67]
Kwashiorkor phenotype^1^	5 (4%)	6 (4%)	4 (6%)	2 (3%)
Altered consciousness^2^	50 (36%)	54 (40%)	29 (43%)	25 (37%)
Restless	44 (32%)	42 (31%)	22 (33%)	20 (30%)
Sunken eyes	136 (99%)	131 (98%)	65 (97%)	66 (99%)
Skin pinch 1-2sec	15 (11%)	12 (9%)	6 (9%)	6 (9%)
Skin pinch >2sec	120 (87%)	122 (91%)	61 (91%)	61 (91%)
Unable to take/retain oral fluids	111 (80%)	104 (78%)	53 (79%)	51 (76%)
Fever (temperature >37.5°C)	49/137 (36%)	45/134 (34%)	16/67 (24%)	29/67 (43%)
Tachypnea (>40 breaths/minute)	34 (25%)	35 (26%)	21 (31%)	14 (21%)
Oxygen saturation (%)	100 (99, 100) [N=137]	100 (98, 100) [N=134]	100 (98, 100) [N=67]	100 (98, 100) [N=67]
Heart rate (beats/minute)	139 (118, 155)	136 (118, 153)	132 (114, 156)	140 (120, 152)
Tachycardia^3^	12 (9%)	14 (10%)	9 (13%)	5 (7%)
Capillary refill time >3seconds	18 (13%)	22 (16%)	14 (21%)	8 (12%)
Weak pulse	23 (17%)	28 (21%)	17 (25%)	11 (16%)
Cold peripheries	18/138 (13%)	23/133 (17%)	12/66 (18%)	11/67 (16%)
Moderate hypotension^4^	42/134 (31%)	34/127 (27%)	18/62 (29%)	16/65 (25%)
WHO Shock^5^	12 (9%)	13 (10%)	8 (12%)	5 (7%)
Indrawing	12/138 (9%)	17/132 (13%)	11/66 (17%)	6/66 (9%)
Deep breathing	15/138 (11%)	11/132 (8%)	6/66 (9%)	5/66 (8%)
Lung crepitations	14/134 (10%)	19/129 (15%)	10/65 (15%)	9/64 (14%)
Bloody diarrhea	30/136 (22%)	24/133 (18%)	14/66 (21%)	10/67 (15%)
History of vomiting	113/137 (82%)	102/133 (77%)	48/66 (73%)	54/67 (81%)
Appetite (1-10 scale) ^6^	3 (2, 5) [N=138]	3 (2, 4) [N=132]	3 (2, 4) [N=65]	3 (2, 5) [N=67]
Still breastfeeding	76/135 (56%)	62/132 (47%)	29/65 (45%)	33/67 (49%)
Currently on nutritional feed^7^	9/135 (7%)	19/128 (15%)	10/63 (16%)	9/65 (14%)
Taken Oral Rehydration Solution in this illness	76/138 (55%)	83/133 (62%)	42/66 (64%)	41/67 (61%)
Admitted to another facility in this illness	67/136 (49%)	74/128 (58%)	36/63 (57%)	38/65 (58%)
Diarrhea in last 6 months	73/137 (53%)	72/132 (55%)	36/65 (55%)	36/67 (54%)
Previous admission for malnutrition	29/136 (21%)	50/127 (39%)	21/62 (34%)	29/65 (45%)
**Laboratory measures**				
Hypoglycemia (glucose <3 mmol/L)	12/135 (9%)	13/131 (10%)	10/64 (16%)	3/67 (4%)
Sodium (mmol/L)	124 (118, 129) [N=131]	124 (119, 130) [N=131]	123 (117, 130) [N=66]	125 (120, 129) [N=65]
Severe hyponatremia (sodium <125 mmol/L)	70/131 (53%)	67/131 (51%)	35/66 (53%)	32/65 (49%)
Potassium (mmol/L)	2.8 (2.0, 3.4) [N=129]	2.5 (2.0, 3.2) [N=129]	2.4 (2.0, 3.3) [N=65]	2.5 (2.0, 3.2) [N=64]
Severe hypokalemia (potassium <2.5 mmol/L)	52/129 (40%)	63/129 (49%)	33/65 (51%)	30/64 (47%)
Chloride (mmol/L)	98 (92, 103) [N=129]	98 (93, 106) [N=130]	98 (92, 105) [N=66]	99 (93, 106) [N=64]
Bicarbonate (mmol/L)	16 (11, 19) [N=55]	13 (10, 17) [N=58]	12 (8, 14) [N=29]	15 (13, 20) [N=29]
HIV positive	0 (0%)	2 (1%)	1 (1%)	1 (1%)
Malaria rapid test positive^8^	25 (18%)	20 (15%)	9 (13%)	11 (16%)
Haemoglobin (g/dL)	11.4 (9.7, 12.6) [N=131]	11.5 (9.6, 12.9) [N=127]	11.6 (9.9, 12.8) [N=64]	11.2 (9.5, 12.9) [N=63]
Severe anemia (Hb < 5 g/dL)	0/131 (0%)	0/127 (0%)	0/64 (0%)	0/63 (0%)
Blood culture positive	4/45 (9%)	8/53 (15%)	3/27 (11%)	5/26 (19%)

1 Bilateral pitting edema of the feet

2 Altered Conscious level – only responding to voice 78 (29%), pain 24 (9%) unconscious 2 (1%)

3 Defined as Heart rate > 160 bpm in children aged <12 months; >120 bpm if aged ≥12 months

4 Defined as Systolic blood pressure 50-75 in children aged <12 months; 60-75 if aged 1-5 years; 70-85 if aged >5 years

5 Defined as having all of the following: cold peripheries with a weak and fast pulse (rate not specified) and a capillary refill time >3 seconds

6 Appetite ranked by parent/guardian on an analogue scale (0=Very poor to 10=very good)

7 Taking ready-to -use therapeutic feed

8 Point of care test indicating the presence of malaria parasites or recent malaria infection in the last 10-14 days.

Note: showing n (col %) or median (IQR).If no denominator is given then it is column total (rapid IV (Plan C)=67; Slow=67; Control (WHO SAM)=138).

**Table 2:** Clinical Management during Hirst 24 hours of Admission

	Control	Liberal (liberal:rapid and liberal:slow combined)	Liberal:rapid intravenous rehydration	Liberal:slow intravenous rehydration	Estimate (liberal vs. control)
Number starting IV fluids within 24 hours of randomization	31/138 (22%)*	133/134 (99%)	66/67 (99%)	67/67 (100%)	
Time to starting fluids (mins)	123 (13-470) [N=31]	14 (13-470) [N=133]	16 (10-28) [N=66]	12 (8-22) [N=67]	
Number of patients in shock at randomization	12/138 (9%)	13/134 (10%)	8/67 (12%)	5/67 (7%)	
Number of patients who received initial bolus (% of those in shock)	12/12 (100%)	7/13 (54%)	7/8 (88%)	0/5 (0%)	
Number receiving treatment for correction of glucose levels during 24 hours from randomization	10/138 (7%)	9/133 (7%)	4/66 (6%)	5/67 (7%)	0.93 (0.36, 2.37)^a^
Number with nasogastric tube inserted during first 24 hours from randomization	126/135 (93%)	82/126 (65%)	43/64 (67%)	39/62 (63%)	0.13 (0.06, 0.30) ^a^
Number with vomiting during first 24 hours from randomization	96/136 (71%)	69/133 (52%)	33/66 (50%)	36/67 (54%)	0.44 (0.27, 0.75) ^a^

¥ Columns show median (IQR) or n/N (%)

* 14 children in WHO SAM arm not in shock at randomization developed shock or had another SAE within 24 hours which was treated with IV fluids. One received a small amount of fluid with thiamine. Three received small amounts of 10% dextrose. One received 5% dextrose 20 hours after randomization.

a Odds ratios (95%CI) estimated with Mantel-Haenszel methods

Note: The widths of confidence intervals for estimates have not been adjusted for multiplicity and should not be used in place of hypothesis testing.

**Table 3:** Primary, Secondary and Other Outcomes.

	Control	Liberal (liberal:rapid and liberal:slow combined)	Liberal:rapid intravenous rehydration	Liberal:slow intravenous rehydration	Estimate (liberal vs. control) (95% CI)
**Primary outcome**					
**Mortality at 96 hours**	11/138 (8%)	9/134 (7%)	5/67 (7%)	4/67 (6%)	1.02 (0.41,2.52)^a^; p=0.69
**Secondary outcomes**					
Mortality at 28 days	17/138 (12%)	14/134 (10%)	8/67 (12%)	6/67 (9%)	0.85 (0.41, 1.78)^b^
Weight change (kg) at Day 3	0.4(0.2) [N=124]	0.5 (0.3) [N=123]	0.5 (0.2) [N=62]	0.5 (0.3) [N=61]	0.1 (0.1, 0.2)^c^
MUAC change (cm) at Day 3	0.2 (0.3) [N=124]	0.3 (0.4) [N=123]	0.3 (0.5) [N=62]	0.3 (0.4) [N=61]	0.1 (0.0, 0.2)^c^
Weight change (kg) at Day 7	0.6(0.3) [N=124]	0.6 (0.4) [N=122]	0.6 (0.4) [N=60]	0.7 (0.4) [N=62]	0.0 (-0.0, 0.1)^c^
MUAC change (cm) at Day 7	0.6 (0.6) [N=124]	0.6 (0.6) [N=122]	0.6 (0.7) [N=60]	0.6 (0.6) [N=62]	0.0 (-0.1, 0.1)^c^
**Safety outcomes**					
Suspected pulmonary edema	0/138 (0%)	0/134 (0%)	0/67 (0%)	0/67 (0%)	
Secondary heart failure	0/138 (0%)	0/134 (0%)	0/67 (0%)	0/67 (0%)	
Urine output at 8 hours (ml)	86 (127) [N=18]	144 (172) [N=18]	112 (109) [N=11]	194 (244) [N=7]	82 (-27, 191)^c^
Time to correction of severe hyponatremia - hazard ratio					1.55 (1.14, 2.09)^†^
Time to correction of severe hypokalemia - hazard ratio					0.82 (0.57, 1.19)^†^
Severe hyponatremia at 8 h	58/126 (45%)	20/128 (16%)	13/64 (20%)	7/64 (11%)	0.23 (0.13, 0.41)^d^
Severe hypokalemia at 8 h	40/126 (31%)	57/128 (45%)	27/64 (43%)	30/64 (47%)	1.79 (1.07, 3.01) ^d^
Change in sodium level at 24 hours from post-IV levels (8 hours)	2.7(6.5) [N=126]	0.7 (6.0) [N=128]	0.4(6.3) [N=64]	1.0 (5.8) [N=64]	0.1 (-1.4, 1.6)^c^
**Other outcomes**					
Ever had an SAE	32/138 (23%)	24/134 (18%)	14/67 (21%)	10/67 (15%)	0.73 (0.40,1.32)^e^
Development of shock	11/138 (9%)	6/134 (5%)	3/67 (5%)	3/67 (5%)	0.55 (0.19, 1.53)^e^
Neurological SAE	0/138 (0%)	1/134 (1%)	0/67 (0%)	1/67 (1%)	
Change in sodium at 8 h (mmol/L)	1.9 (6.0) [N=126]	7.5 (5.0) [N=128]	7.4(5.3) [N=64]	7.5 (4.7) [N=64]	5.7 (4.5, 7.0)^c^
Hypernatremia at 8 h	2/126 (2%)	2/128 (2%)	0/64 (0%)	2/64 (3%)	1.01 (0.14, 7.29) ^d^
Change in potassium (mmol/L) at 8 h	0.2 (0.8) [N=126]	-0.1 (0.9) [N=128]	-0.1 (1.0) [N=64]	-0.1 (0.8) [N=64]	-0.3 (-0.5, -0.2)^c^
Change in sodium at 24 h	4.4 (7.4) [N=126]	8.2 (7.2) [N=127]	7.8 (7.8) [N=64]	8.6(6.6) N=63]	4.1 (2.6, 5.7)^c^
Severe hyponatremia at 24 h	35/126 (27%)	21/127 (17%)	13/64 (20%)	8/63 (13%)	0.53 (0.29, 0.98)^d^
Hypernatremia at 24 hours	3/126 (2%)	6/127 (5%)	3/64 (5%)	3/63 (5%)	2.08 (0.51, 8.51)
Severe hypokalemia at 24 h	26/126 (20%)	36/127 (29%)	17/64 (27%)	19/63 (30%)	1.59 (0.89, 2.83) ^d^
Change in potassium at 24 h	0.6 (1.0) [N=126]	0.2 (0.9) [N=127]	0.3 (0.9) [N=64]	0.1 (0.8) [N=63]	-0.4 (-0.6, -0.2)^c^

¥ Columns show mean (sd) or n/N(%)

a Risk ratio (95%CI), Mantel-Haenszel adjustment for site.

b Hazard ratio (95%CI) (Cox regression) (PH assumption tested p=0.89, all covariates p>0.22)

c Difference in means (95% CI)

d Odds ratio (logistic regression) (95%CI)

e Odds ratio

† subhazard ratio, with death as a competing risk.

Note: The widths of confidence intervals for estimates have not been adjusted for multiplicity and should not be used in place of hypothesis testing.
