# Supplementary material for: Intravenous Rehydration for Severe Acute Malnutrition with Gastroenteritis
Source: N Engl J Med. Author manuscript; Available in PMC 2025 Oct 2. (PMC7617792; doi:10.1056/NEJMoa2505752)
Supplement: Supplement [file EMS205900-supplement-Supplement.pdf]

SUPPLEMENTARY APPENDIX

Table of Contents

List of Investigators and Committee Members 3

List of Investigators 3

List of Committee Members 4

Supplementary Methods 5

Trial Sites 6

(a) Trial Design: Hypothesis and Objective 7

(b) Inclusion/Exclusion Criteria from Main Trial Protocol 7

(c) Further Details of Allocation 8

(d) Further Details of Endpoint Ascertainment 8

(e) Sample Size Calculation 9

(f) Analysis Methods for Multiple Imputation 10

(g) Ethics Approvals 10

(h) Administration of Trial Interventions 10

(i) Second Randomization 12

(j) Protocol Versions 12

(k) Interim Analyses 13

Supplementary Tables and Figures 15

Figure S1: Full Trial Schema 15

Figure S2: Trial Flow 16

Figure S3: Enrollment by site 17

Figure S4: Kaplan Meier Plot of Mortality at 28 Days: Control, Liberal:rapid, and Liberal:slow. 17

Figure S5: Time to Correction of Hyponatremia: Control vs. Liberal 18

Figure S6: Time to Correction of Hyponatremia: Control, Liberal:rapid and Liberal:slow. 18

Figure S7: Time to Correction of Hypokalemia: Control vs. Liberal 19

Figure S8: Time to Correction of Hypokalemia: Control, Liberal:rapid and Liberal:slow. 19

Figure S9: Residual Plots From All Linear Regression Models 20

Table S1: Representativeness of Study Population 21

Table S2: Intravenous Fluid Administration Rates for Liberal:rapid arm 24

Table S3: Intravenous Fluid Administration Rates for Liberal:slow arm 25

Table S4: Intravenous Fluid Administration Rates for Boluses Given to Those in Shock in Control Arm 25

Table S5: Details of Rehydration Solutions 26
Table S6: Further Detail on Adherence to Intravenous Rehydration Strategy 27
Table S7: Other Outcomes from Statistical Analysis Plan: Liberal vs. Control; Liberal:rapid vs. Control and Liberal:slow vs. Control. 28
Table S8: Primary, Secondary and Other Outcomes: Liberal:rapid vs. Control and Liberal:slow vs. Control Separately 29
Table S9: Subgroup Analysis for Primary Endpoint of Mortality at 96 Hours 30
Table S10: Safety Events 31
Table S11: List of SAEs 32
Table S12: Secondary Outcomes for the Liberal vs. Control Comparison Estimated with Multiple Imputation 34

References 35

List of Investigators and Committee Members

List of Investigators

Name	Position	Affiliation	Country
Phyles Maitha	Trial administrator	Kilifi Clinical Trials Facility KEMRI Wellcome Trust Research Programme, Kilifi	Kenya
Nchafatso Obonyo	Paediatrician	KEMRI Wellcome Trust Research Programme, Kilifi	Kenya
Jay Berkley	Paediatrician	KEMRI Wellcome Trust Research Programme, Kilifi	Kenya
Kirsty Houston	Paediatrician	KEMRI Wellcome Trust Research Programme, Kilifi	Kenya
Victor Bandika	Consultant Paediatrian/ (Site-PI)	Coast General Teaching and Referral Hospital	Kenya
Laura Mwalekwa	Clinical Officer	Coast General Teaching and Referral Hospital	Kenya
George Passi	Medical Officer	Mbale Regional Referral Hospital	Uganda
Moses Oluput Olupot	Study coordinator	Mbale Regional Referral Hospital	Uganda
Charles B Okalebo	Pharmacist	Mbale Regional Referral Hospital	Uganda
Rita Muhindo	Head of laboratory	Mbale Regional Referral Hospital	Uganda
Grace Abongo	Data Manager	Mbale Regional Referral Hospital	Uganda
Margret Nakuya	Medical Officer	Soroti Regional Referral Hospital	Uganda
Hastings C Onu	Epidemiologist	Médecins Sans Frontières (MSF), Abuja	Nigeria
Mohammed Ashir Garba	Clinician	Faculty of Medicine, University of Maiduguri Teaching Hospital, Maiduguri	
Mark Lee	Paediatric Advisor	Médecins Sans Frontières, Geneva	Switzerland
Grace Mambula	Pharmacovigilance Specialist	Epicentre, Paris,	France
Johanna Thomson	Paediatric Working Group Lead	Médecins Sans Frontières, Paris	France

List of Committee Members

Trial Steering Committee

Professor Elizabeth Molyneux, OBE (Chairman): College of Medicine, Blantyre, Malawi

Dr Jane Crawley University of Oxford, United Kingdom

Dr Irene Lubega Department of Paediatrics Makerere University College of Health Sciences, Uganda

Professor William Macharia Department of Paediatrics and Child Health, Aga Khan Hospital, Nairobi, Kenya.

Independent Data Monitoring Committee

Prof. Timothy Peto (Chair) University of Oxford, Oxford, United Kingdom

Dr. Jennifer Thompson (statistician) (for 3 reviews), London School of Hygiene and Tropical Medicine, London, United Kingdom

Dr Nuredin Mohammed (statistician) (for 1 review), MRC Unit at The Gambia, London School of Hygiene and Tropical Medicine, The Gambia.

Professor Dr. Edison Mworozi Department of Paediatrics, Mulago Hospital, Kampala, Uganda

Professor Philippa Musoke Makerere University College of Health Sciences, Kampala, Uganda.

Supplementary Methods

Relevant Guidelines (with page references regarding rehydration)

- WHO ETAT guideline Page 45 general, Page 47 severe malnutrition https://iris.who.int/bitstream/handle/10665/43386/9241546875_eng.pdf

- MSF guidelines Chapter 5 Page 199 https://medicalguidelines.msf.org/sites/default/files/2024-10/MSF_Paediatric%20care_2024.pdf

- Kenya National Guidelines Page 36 (general guideline for gastroenteritis) p 44 specific guideline http://guidelines.health.go.ke/#/category/55/478/meta

- WHO Guideline: Updates on the management of severe acute malnutrition in infants and children. Geneva WHO, 2013. Chapter 6 pages 46-49. (http://www.who.int/nutrition/publications/guidelines/updates_management_SAM_infantandchildren/en/).

  "Children with severe acute malnutrition who present with some dehydration or severe dehydration *but who are not shocked* should be rehydrated slowly, either orally or by nasogastric tube, using oral rehydration solution for malnourished children (5–10 mL/kg/h up to a maximum of 12 h)".
  (strong recommendation, low quality evidence)

- WHO guideline on the prevention and management of wasting and nutritional oedema (acute malnutrition) in infants and children under 5 years. Geneva: World Health Organization, 2023.

  Management of severe dehydration due to diarrhea was not reviewed

There are references to standard of care in observational studies of severe malnutrition which have indicated they follow national/international guidelines for rehydration[1-3]. Mortality rates for children with severe dehydration in these studies ranged from 27%-41%.

Trial Sites

Kilifi County Hospital, Kenya
Annual rates of pediatric and neonatal admission to KCH are 4500. KCH has a general pediatric ward (staffed by county clinical staff) and a 5-bedded high dependency unit which frequently has a 200% bed occupancy (staffed by dedicated research teams 1 doctor) and a neonatal side ward with capacity for 9 incubators/cots (staffed by clinical research teams 1 doctor and 3 nurses per shift)

Coast General Hospital, Kenya
This is a government hospital with a general pediatric ward (staffed by county clinical staff) and has no dedicated high dependency care unit.

Mbale Regional Referral Hospital, Uganda
Annual rates of pediatric and neonatal admission to MRRH are 19000. MRRH has a general pediatric ward (staffed by county clinical staff) and has no dedicated high dependency care unit.

Soroti Regional Referral Hospital, Uganda
Annual rates of pediatric and neonatal admission to SRRH are 8500. SRRH has a general pediatric ward (staffed by county clinical staff) it has no dedicated high dependency care unit.

Magaria District Hospital, Niger
Magaria District Hospital is a secondary-level hospital managed by the Ministry of Public Health. It serves a largely rural catchment area (population 931 254) in south-central Niger, and its pediatric and nutrition wards are supported by Médecins Sans Frontières. In 2024, 10722 children <5 years were admitted as inpatients in the pediatrics department, 11306 to inpatient therapeutic feeding centers, and 28012 to malaria-specific units during the seasonal malaria peak.

Nilefa Kiji MSF Hospital, Maiduguri, Nigeria

Nilefa Keji Hospital, operated by Médecins Sans Frontières Operational Centre Brussels (MSF-OCB), in Borno State, Nigeria (population ~6.11 million). It provides care to children with acute malnutrition. In 2024, the Inpatient Therapeutic Feeding Centre (ITFC) admitted 16,280 patients, with 12.4% requiring ICU care, with average Bed Occupancy Rate (BOR) of 87% but reached 167% during peak season (August to October). The mortality rate was approximately 4%. The ITFC has an Emergency Room (ER), Intensive Care Unit (ICU) of 20 beds (expanded to 40 during highest peak of August-October), phase I and transition phase beds (total 120 bed) and an isolation unit (20 beds, up to 38 beds during peak). Overall bed capacity varies seasonally from 200 to 400 beds. Staffing ranges from 400 in the low season to 800 in peak periods. The GASTROSAM trial ward was a nine-bed unit in the ER Extension that had dedicated staff of 4 doctors, 8 nurses, 4 nutrition assistants, a counsellor, and a health promoter.

(a) Trial Design: Hypothesis and Objective

We had two hypothesis which relate to i) immediate management of severe dehydration and ii) Type of oral rehydration solution to use in both severe dehydration and moderate dehydration

For children with SAM with severe dehydration we hypothesized that standard intravenous regime WHO PlanC (100mls/kg over 3-5 hours) used for non-SAM gastroenteritis with severe dehydration will result in better outcomes than the current very conservative SAM rehydration recommendations. In addition, we propose that the rate of rehydration may be critical and hypothesize that 100mls/kg over 8 hours in SAM children will result in fewer fluid related adverse effects than rapid WHO Plan C guideline.

We also propose that standard oral rehydration solutions (ORS) may be equally as effective with fewer side effects than low-sodium Rehydration Solution for Malnutrition (ReSoMal).

Major Objectives:

To compare the rate and volume of rehydration in children with signs of severe dehydration (see study population) secondary to gastroenteritis on a primary endpoint of 96-hour mortality:

(i) Current standard WHO rehydration protocol Plan C usually used in non-SAM children including boluses for shock
(ii) A slower rehydration regimen using the same total volume (100ml/kg) over 8 hours, irrespective of age and no boluses for shock
(iii) The current WHO restrictive intravenous rehydration strategy for SAM children.

In children with diarrhea complicated by moderate or 'some' dehydration (see study population) and as follow-on rehydration post-intravenous rehydration in those with severe dehydration whether oral rehydration with

(i) WHO standard oral rehydration solution (ORS) for non-SAM[21] is safer and results in less hyponatremia and better outcomes compared to
(ii) current recommendation advocating low sodium (ReSoMal) ORS[4]

(b) Inclusion/Exclusion Criteria from Main Trial Protocol

Inclusion criteria:
Children hospitalized with SAM criteria (defined as any of: mid-upper arm circumference (MUAC) <11.5cm, WHZ <-3SD or kwashiorkor)[4], aged 6 months to 12 years, with

gastroenteritis (> 3 loose stools/24 hours):

Severe Dehydration: Stratum A
Signs of severe dehydration (as per WHO definition two or more of: unable to drink or AVPU <A, sunken eyes or reduced skin pinch (>2seconds) or an inability to take or retain oral fluids), with or without shock. Shock defined by the recent 2016 WHO ETAT criteria; a patient with all of the following: cold peripheries with a weak and fast pulse (rate not specified) and a capillary refill time >3 seconds[4].

Moderate/Some Dehydration: Stratum B
Defined as two or more of restlessness or irritable, thirsty, sunken eyes or skin pinch goes back slowly (<=2 s).

Exclusion Criteria for All:

Diarrhea lasting more than 14-days
Known congenital or rheumatic heart disease
Refusal of consent

(c) Further Details of Allocation

Randomisation lists were generated separately for each stratum and kept at the MRC CTU at UCL, London. Opaque sealed envelopes containing the randomized allocation(s) were prepared at the Clinical trials facility, KWTRP, Kilifi and shipped to the clinical trial sites. The envelope contained the actual allocation for intravenous and oral strategies which was visible only once opened. The cards were numbered consecutively within each stratum and opened in numerical order. This method was used to ensure that acutely unwell children could be treated immediately at the emergency ward, without having to rely on a computer or the internet to obtain a randomized allocation.

The allocation ratio was 2:1:1 so that we could combine the two liberal strategies together and for this total to match those in the standard of care arm. This would ensure the power for this comparison, given expected mortality rates, balanced against what was realistic for recruitment in these settings

(d) Further Details of Endpoint Ascertainment

SAEs were defined following the International Committee for Harmonisation as events which led to death, were life-threatening, caused or prolonged hospitalization (excluding elective procedures), caused permanent disability, or were other medical conditions or with a real, not hypothetical risk of one of the previous categories.

At each clinical review the clinicians were asked to observe for solicited SAEs which included shock and clinical signs of fluid overload. Severe adverse events used the standardized definitions and internal review processes of these events will be carried out using the same criteria as were used in the FEAST trial[5]. All relevant adverse events are reported in the case report form and SAE form. The reporting procedure was captured within the safety reporting SOP.

(e) Sample Size Calculation

This trial started as a Phase II trial with a focus on safety (with a urine output endpoint), but was then after COVID was extended to look at mortality with the joining of MSF sites who were able to recruit rapidly and to monitor children very closely (given concerns about fluid overload events).

Below are details taken from the Protocols regarding sample size calculations.

In version 1.0 the GASTROSAM sample size calculations for both randomisations were based on studies conducted in children with malnutritionand diarrhea. For the primary endpoint of urine output for the GASTROSAM A stratum this was based on two African studies have reported on urine output (a surrogate for rehydration and perfusionstatus) in response to intravenous rehydration. Akech *et al*[6] reported persistence of oliguria (urine output<1ml/Kg/hour) at 8 hours was more common in those receiving the WHO regimen with hypotonic solution (mean volume 30ml/Kg, with 9/22 (41%) as oliguric) vs. children receiving isotonic Ringers Lactate (mean volume39ml/Kg, 3/25 (12%) as oliguric), p=0.05[12]). In the second study using RL for rehydration oliguria was present in 3/11 (27%) on WHO regime vs. 2/9 (22%) standard rehydration fluid-resuscitation[7].

For Version 3.0 we reviewed the literature on mortality which was informed by a physiological observational study investigating liberal rehydration versus WHO standard of care (the AFRIM study)[7]. Overall, mortality in the **WHO arm was 9/11 (81.8%) to Day 28** and of these deaths **7/11 had occurred by 96-hours** (64% mortality; unpublished data (Obonyo)). We therefore felt that a primary endpoint of 96 hours for this current trial of intravenous rehydration strategies was suitable and also that we would be able to achieve complete retention in the trial to this endpoint (96 hours survivorship). Later time points would increase the risk of missing data for the primary endpoint due to the very wide catchment area of the hospitals and the expected difficulty of participants returning to hospitals.

On the basis of the available data, predominately from the AFRIM study, we estimated a slightly lower mortality of 58 % in the WHO arm (as mortality is often lower in clinical trials) a 30% relative reduction in mortality rate at 96 hours to 41% in the combined liberal arms would have a power of 80% and a 2-sided test with alpha 0.05. This would require a higher number in the intravenous rehydration group 272 (or an extra 136 children from the original

sample size).

Based on these data we calculated that for the Stratum A randomisation, 272 children would give 80% power (with 1:1:2 randomisation), with 68 to the rapid WHO Plan C arm, 68 to slow rehydration arm (100mls/kg over 8 hours) and 136 to the WHO SAM arm to show a 30% lower 96 hour mortality in the liberal strategies compared to the WHO SAM arm. All children in Stratum A will also be randomised to ORS strategies.

More details on the sample size calculation and numbers for the ORS randomisation are in the current version of the trial protocol.

(f) Analysis Methods for Multiple Imputation

Multiple imputation using chained equations was performed separately for each randomised group in the factorial, including all trial participants. Separate imputation models were used for biochemistry and anthropometric outcomes. In the biochemistry model, sodium at 8 hours, 24 hours, and day 7 were imputed with linear regression and potassium values were imputed with predictive mean matching. Severe hyponatremia, severe hypokalemia and hypernatremia were passively imputed. In the anthropometry model, weight and MUAC at day 0, day 3 and day 7, and their z-scores were imputed via linear regression. Both imputation models included site, age, and death before each time point as predictors, and produced 30 imputed datasets. Values that were missing due to death were imputed and then set to missing before analysis. Weight and MUAC z-scores that were missing due to age, or being extreme outliers, were set to missing in the imputations as well. Boxplots were used to visually compare observed and imputed data. Parameter estimates from the imputed models were combined using Rubin's rules.

(g) Ethics Approvals

The national ethics committees of Kenya, Niger and Nigeria approved the protocol, as well as Mbale Regional Referral Hospital, Uganda. In addition,he ethics committees of Imperial College London, UK, and MSF approved the protocol.

(h) Administration of Trial Interventions

Details of Trial Rehydration Strategies

- **Rapid liberal rehydration (liberal:rapid) (experimental)**: Rapid iv rehydration as per WHO Plan C (recommended for children who without SAM): a two-step rehydration treatment giving 100mls/kg Ringers Lactate (RL) over 3-6 hours according to age and including

boluses (20mls/kg) for those with shock.

Children in shock at admission in this arm:

Ringers lactate or normal (0.9%) saline 20mls/kg given over 15 minutes. If the child remains in shock the one repeat bolus permissible. Then step 1 below is left out and rehydration proceeds straight to step 2 (see above for rates)

Children who are less than one year

Step 1: Ringer's lactate or 5% Dextrose/Ringers at 30mls/kg over 1 hour
Step 2: 70mls/kg over 5 hours

Children who are older than one year

Step 1: Ringer's lactate/5% Dextrose/Ringers at 30mls/kg over 30 minutes
Step 2: 70mls/kg over 2 hours and 30 minutes

This strategy was followed by oral rehydration (see below). Children were permitted to start oral rehydration, if thirsty before the end of intravenous rehydration.

Slow liberal rehydration (liberal:slow) (experimental): All children (irrespective of age) randomized to this strategy received Ringer's lactate or 5% Dextrose/Ringers (Darrows) 100mls/kg over 8 hours given at the same rate throughout the 8-hour period. Even if the child is in shock – no boluses are permitted in this strategy.
This strategy was followed by oral rehydration (see below). Children were permitted to start oral rehydration, if thirsty before the end of intravenous rehydration.

WHO recommended rehydration for children with SAM (control) (standard of care):
Children in this arms proceeded directly to administration of Oral rehydration solution (ORS) as indicated in by randomisation. ORS was to be taken by mouth or by nasogastric tube if the child could not take or tolerate oral fluids (see rates below).

Children in this arm (irrespective of age) who were in shock at admission or who subsequently developed shock received an intravenous bolus of HSD/5% dextrose or Ringer's lactate/5% dextrose or 0.45 saline/5% dextrose at 15mls/kg over 1 hour. If shock persisted one repeat bolus was permitted of infusion 15mls/kg over 1 hour then switched to oral or nasogastric rehydration with ORS. If there was no improvement after two hours of intravenous fluid therapy the WHO protocol recommends that children start on intravenous maintenance fluids at 4mls/kg/hour whilst awaiting a blood transfusion (whole blood) at 10mls/kg over at least 3 hours (or packed calls (5mls/kg).

Infusion Rates for Intravenous Fluids and Monitoring: All Arms

Intravenous fluids were administered using 150 ml gauged burettes plus or minus infusion pumps. Infusion rates for intravenous fluids varied depending on the child's age, weight, and randomized treatment. See Supplementary Tables S1,S2 and S3 for all rates and Table S4 for solution details.

(i) Second Randomization

All children with severe dehydration require oral rehydration throughout admission for one going losses. We therefore chose to do this by randomisation (see Trial Schema Fig S1).

The additional factorial randomization for type of oral rehydration was between

- **WHO SAM-recommended low-sodium ReSoMal (control)**

  All children (irrespective of age) who are randomized to this arm will receive 5mls/kg of ReSoMal every 30minutes for the first 2 hours then 5-10 mls/kg/hour ReSoMal should be given in alternate hours with F-75 up to a maximum of 10 hours.

  Or

- **Standard WHO ORS as recommended for non-SAM (experimental)**

  All children (irrespective of age) who are randomized to this arm should receive ORS 75mls/kg orally over 4 hours. After this assessment and classification of the child, if the child improved, this should then be followed by ORS 10mls/kg after each bout of diarrhea. The ORS can be given in alternate hours with F-75.

If cholera was suspected this randomization was ignored (if to ReSoMal) and children received standard WHO ORS as per WHO cholera recommendations for SAM.

(j) Protocol Versions

Version 1 to Version 1.2

Version 1.0 was the original protocol submitted for ethical approval to Imperial College Research Ethics Committee in February 2018 and approved (20th March) following additional background information included in the Protocol (version 1.1: 5th June 2019). Further clarifications were requested following review by Mbale Regional Referral Hospital Research Ethics, Uganda Version 1.2 was approved by MRRH-REC on June 14th 2019.

Version 1.2 to Version 2.0

Minor Amendment- additional staff members added to replace departing staff (Sept 23rd 2019) prior to any enrollment.

Version 2.0 to 2.2

Major changes:

1) Dropping of the Mulago site, Kampala, Uganda
2) Addition of a second site in Kenya, Coast General Hospital, Mombasa

Minor changes

1) Changes in members of the investigator teams (removal of Mulago site investigators and addition of new investigators for Kilifi and Mombasa sites; change of Imperial administrator)

Version 3.0 (dated 25th November 2022)

Major Changes

1) Addition of an extra study sites in Niger and Nigeria and addition of the 2 new study co-investigators

Owing to slow enrollment in both Uganda and Kenya, Medicines Sans Frontières (MSF) were approached to join the trial. Three MSF centres were selected, one in Nigeria and two in Niger (with three additional principal investigators for these sites). It was anticipated that the main site would be Magaria with a reserve site in case of slow enrollment. The decision to involve MSF, who have a substantial experience in managing children with severe malnutrition and have large case numbers, was endorsed by the independent Trial Steering Committee (TSC) and subsequently approved by the funders (MRC UKRI). Pragmatically we agreed that 2-3 patient per day would be enrolled at these sites in order to ensure quality of trial conduct.

2) Change of primary endpoint for intravenous rehydration stratum and increase in sample size
A concern raised by the investigators and TSC was whether, if the trial were to show significantly better survival for those receiving liberal rehydration, this would be sufficient to advocate for guideline change given that mortality was a secondary endpoint in protocol Version 2.1, and urinary output at 8 hours was the primary endpoint. The primary endpoint was therefore changed to mortality at 96 hours post randomisation; and a sample size to reflect this new endpoint was calculated.
3) The change in primary endpoint and modest increase in sample size was approved by the TSC and the funders (MRC UKRI).

Minor Changes

4) Clarification of follow up at Day 7 and Day 28 (post enrollment/randomisation) rather than post discharge as incorrectly stated in the study protocol. There were inconsistencies in the protocol where Day 7 and Day 28 (post enrollment) were indicated on the Tables representing study procedures but in the text this was written as Day 7 and Day 28 (post discharge). The two secondary endpoints that this would affect were weight and MUAC at Day 7. For patients already enrolled in the trial we reviewed the data gathered in the trial to date and were reassured that these outcomes could be obtained from the daily weight and MUAC measures for most where they were discharged after 7 days.
5) Clarification that sodium levels at 24 hours and 8 hours were post-randomisation rather than completion of iv treatment (post-iv) (Summary and Page 20)
6) Addition of Day 3 anthropometry to record post rehydration weight/MUAC since some children were discharged before Day 7 (Summary, Table and Page 21)
7) Simplification of additional study procedures, especially relating to urine collection and sample processing where facilities are not available.
8) Many additional procedures focused on providing data for physiological measures (eg plasma and urine osmolarity, endotoxin and other biomarkers) were indicated as optional and only where laboratory facilities permit.
9) All safety endpoints were to be monitored as per the original protocol.

(k) Interim Analyses

The DMC Charter outlined the responsibilities of the DMC and stated: The DMC should inform the Chair of the TSC if, in their view, the results are likely to convince a broad range of clinicians, including those supporting the trial and the general clinical community, that, on

balance, one trial arm is clearly indicated or contraindicated for all participants or a particular category of participants. It is important that the implications (e.g. ethical, statistical, practical, and financial) for the trial be considered before any recommendation is made.
The statistical implications would be based on the Haybittle-Peto rule, even though not explicitly stated, as this is used in practice by trials run by this group with the same DMC. As this rule was implicitly being used then the number of interim analyses was not fixed in advance, as it has a trivial impact on the final alpha/Type I error.

The DMC met four times. The trial statisticians prepared a confidential report including interim analyses as outlined in the statistical analysis plan (page 12) that was circulated only to the DMC committee members. The meeting dates and the DMC conclusions are given below. These conclusions were reported to the TSC and trial investigators.

11th June 2020:

We have no safety concerns and see no reason for any of the trial arms to stop, or be modified in any way.
We appreciated the detail given in the open session regarding the rational and progress of the trial including challenges in recruitment. We understand the difficulties, and note the efforts that were made to increase recruitment prior to the trial being paused due to COVID19.

12th December 2022:

We have no safety concerns and see no reason for any of the trial arms to stop, or be modified in any way.
We appreciated the detail given in the open session regarding the rational and progress of the trial including details of the proposed changes. We are pleased that the trial is expanding to new sites to ensure continued recruitment.

23rd May 2024:

We have no safety concerns and see no reason for any of the trial arms to stop, or be modified in any way.
We are pleased that MSF has supported this trial and that there is an increased number of sites open. We note high levels of follow up and hope for continuation of current good levels of recruitment.

5th September 2024:

We have no safety concerns and see no reason for any of the trial arms to stop or be modified in any way.
We congratulate the site teams on their current recruitment levels and good retention rates.

Supplementary Tables and Figures

Figure S1: Full Trial Schema

Children enrolled into Stratum B do not contribute to analyses presented in this paper.

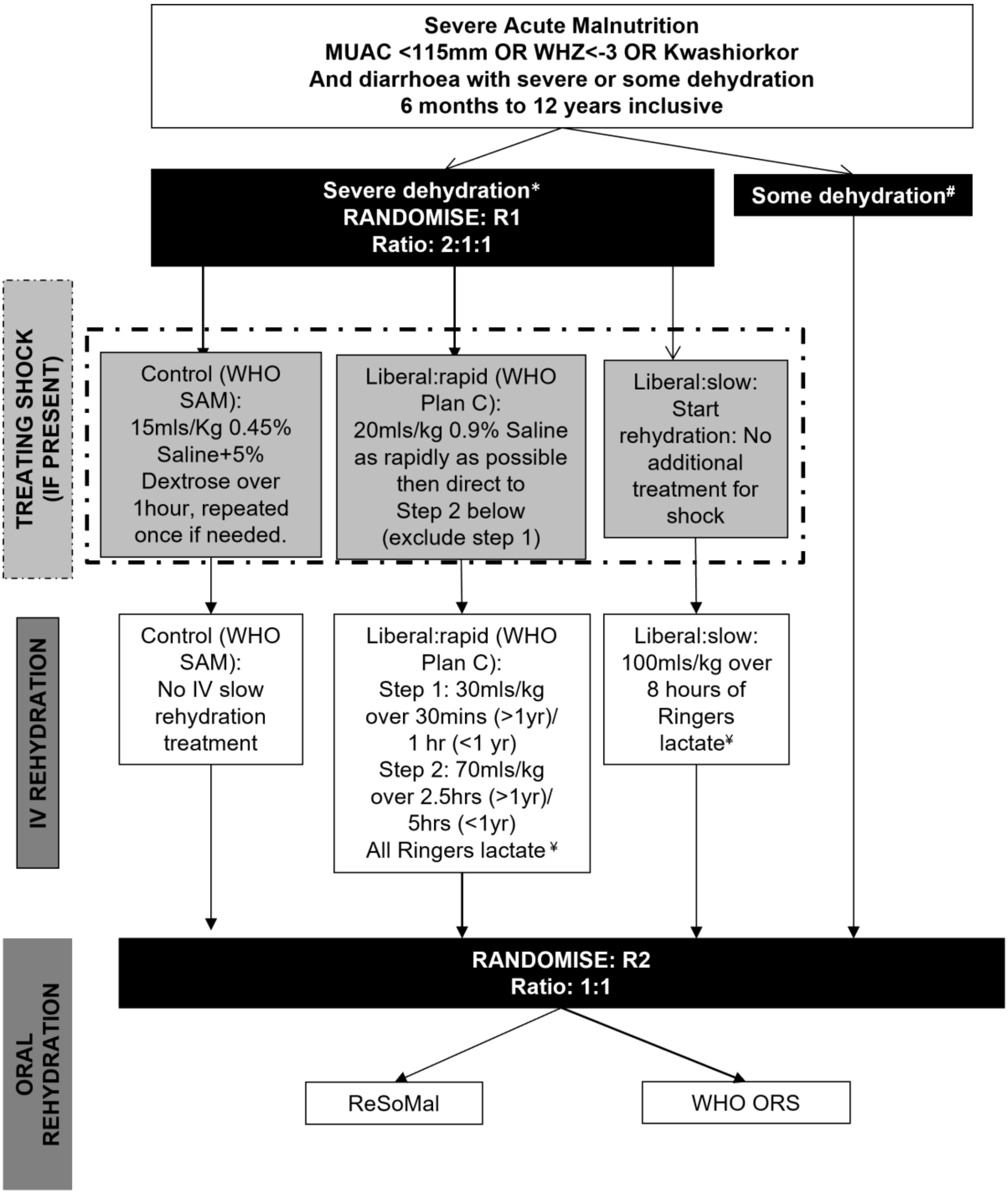

Assessment of severity of dehydration as per WHO 2013 Pocketbook
*All children receiving IV fluids for severe dehydration (R1) will also be randomised as per oral rehydration (R2).
#All children who present with 'some' dehydration will be randomised as per R2. If they go on to develop severe dehydration during admission, they will follow current WHO SAM guidelines.
¥Ringer's lactate/5% Dextrose can be used in place of Ringer's lactate

Figure S2: Trial Flow

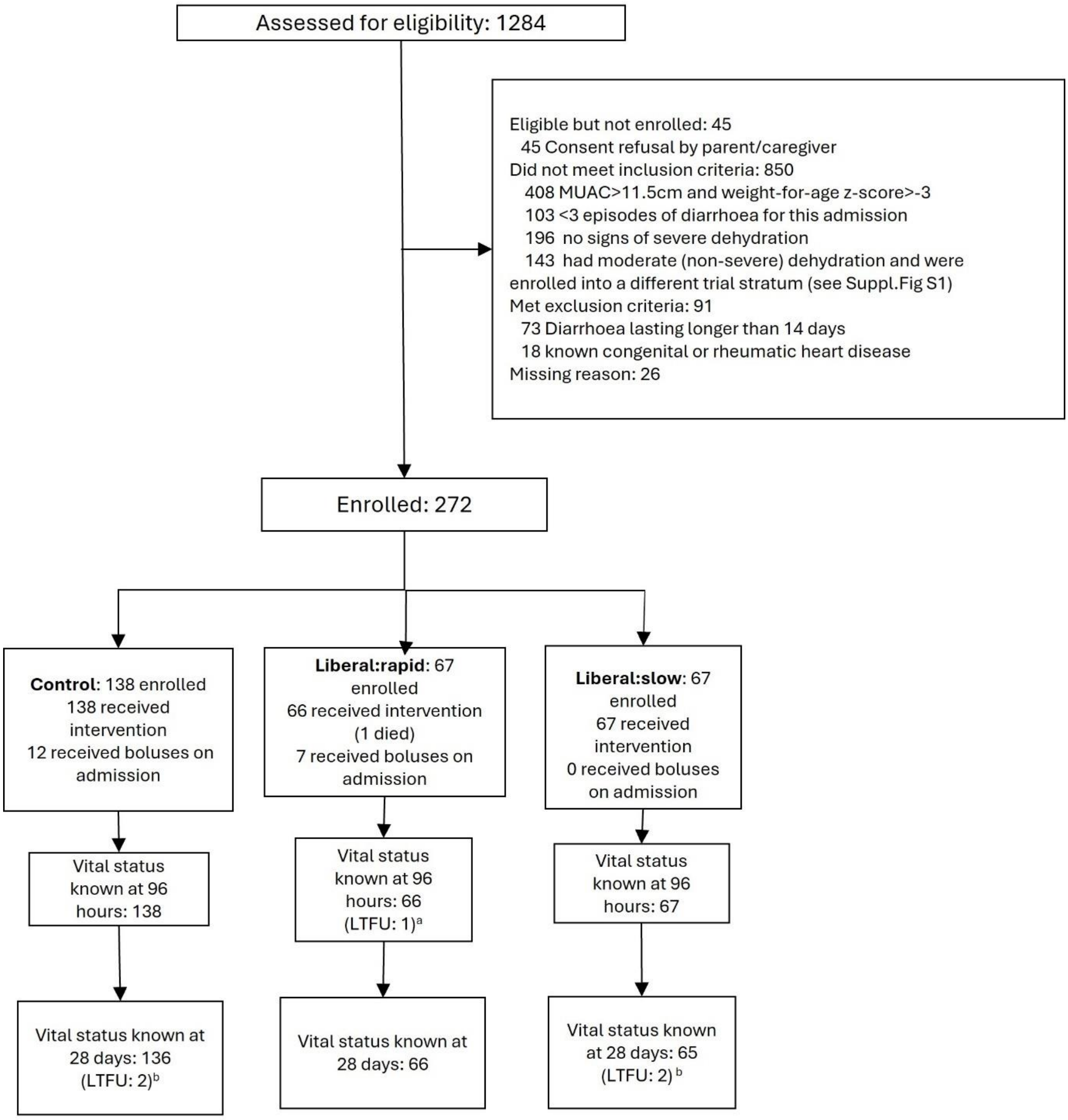

[a]Child assumed alive for estimate of risk ratio at 96 hours.
[b]Children censored at last known alive date for 28 day mortality secondary endpoint.

Figure S3: Enrollment by site

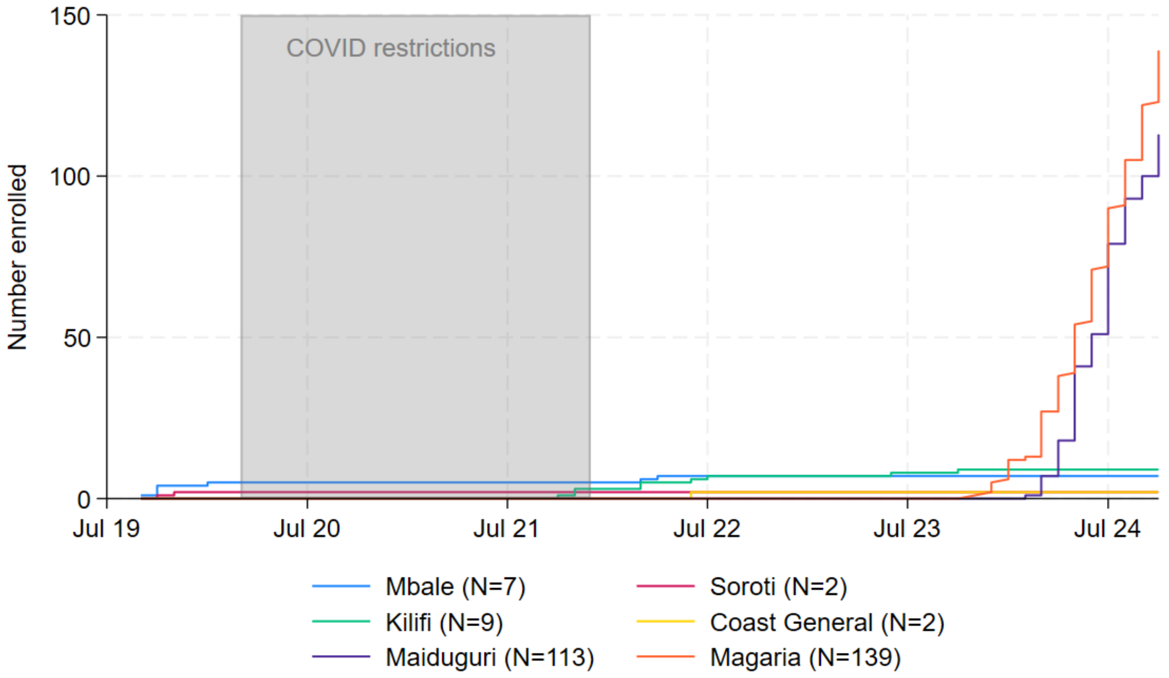

NB: The whole trial was very impacted by the COVID epidemic and restrictions were severe in Uganda such that children were not accessing care in hospitals. Concern was particularly high in Kenya and Uganda affecting equipoise for recruitment into the trial. The trial did not restart after COVID in Uganda (Mbale and Soroti sites).

Figure S4: Kaplan Meier Plot of Mortality at 28 Days: Control, Liberal:rapid, and Liberal:slow.

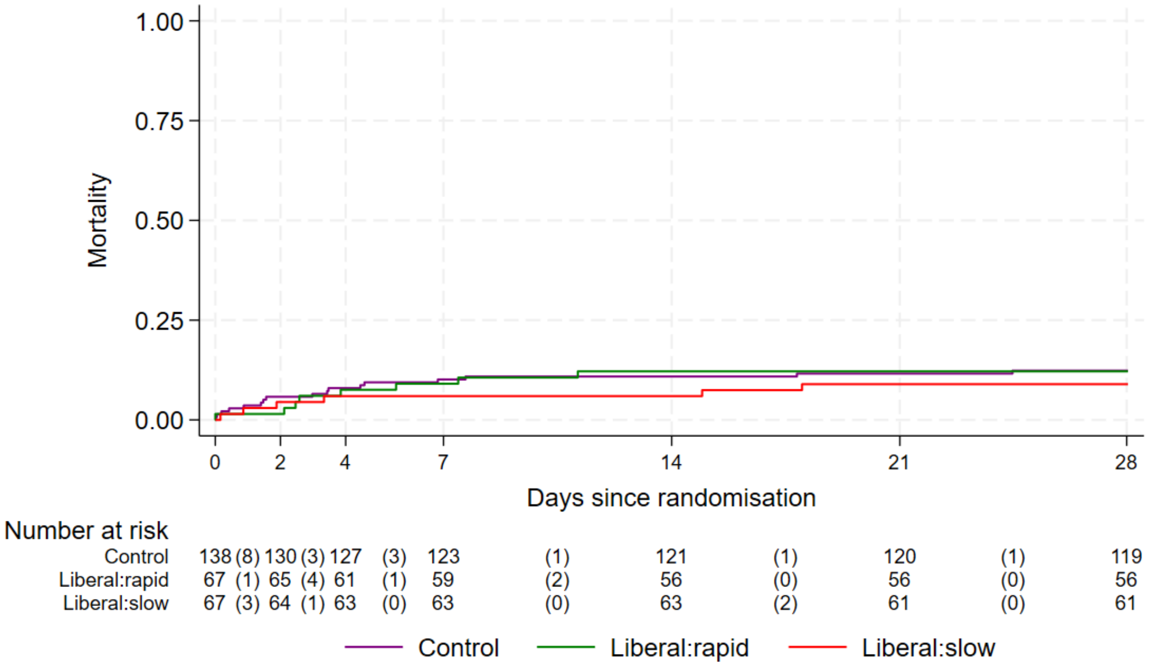

Figure S5: Time to Correction of Hyponatremia: Control vs. Liberal

Cumulative incidence graph estimated from competing risks model with death as a competing risk. Restricted to those with hyponatremia (sodium<125mmol/L) at baseline (N=137).

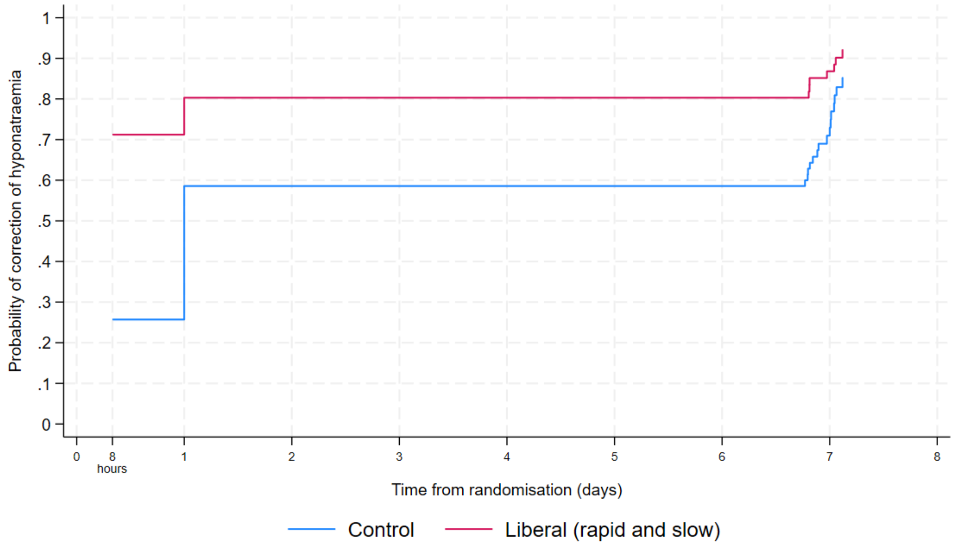

Figure S6: Time to Correction of Hyponatremia: Control, Liberal:rapid and Liberal:slow.

Cumulative incidence graph estimated from competing risks model with death as a competing risk. Restricted to those with hyponatremia (sodium<125mmol/L) at baseline (N=137).

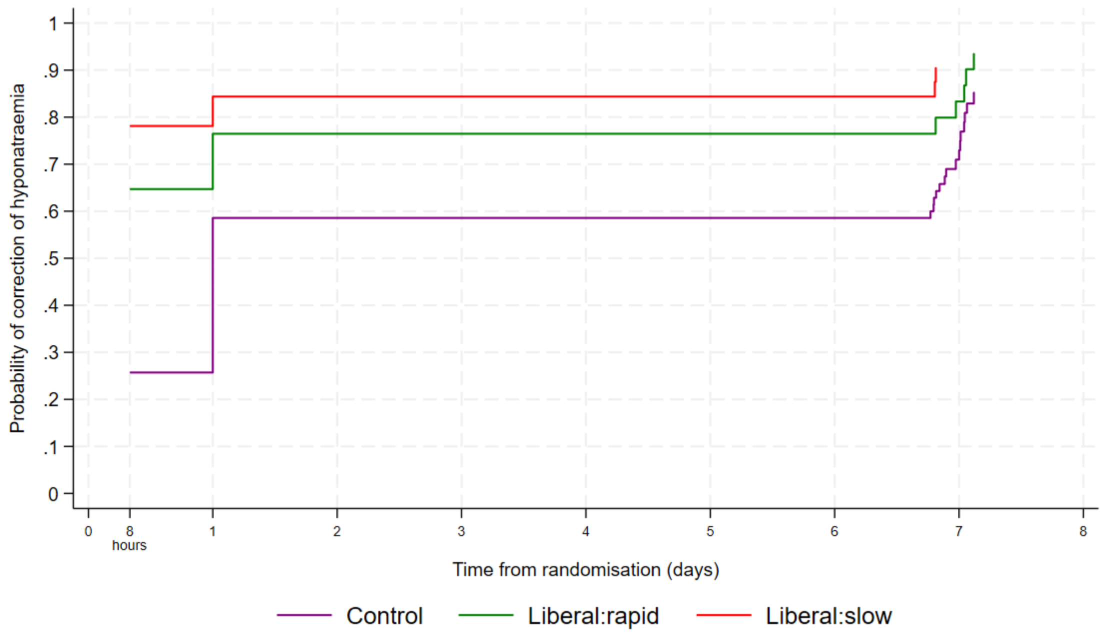

Figure S7: Time to Correction of Hypokalemia: Control vs. Liberal

Cumulative incidence graph estimated from competing risks model with death as a competing risk. Restricted to those with hypokalemia (potassium<2.5mmol/L) at baseline (N=115).

.

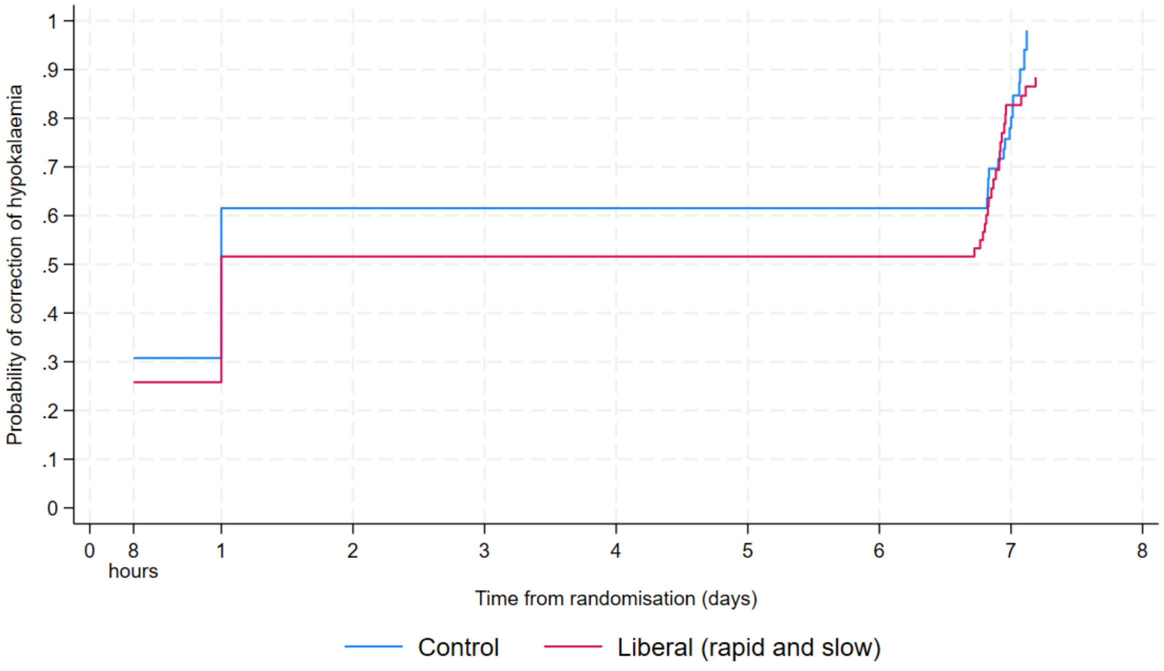

Figure S8: Time to Correction of Hypokalemia: Control, Liberal:rapid and Liberal:slow

Cumulative incidence graph estimated from competing risks model with death as a competing risk. Restricted to those with hypokalemia (potassium<2.5mmol/L) at baseline (N=115).

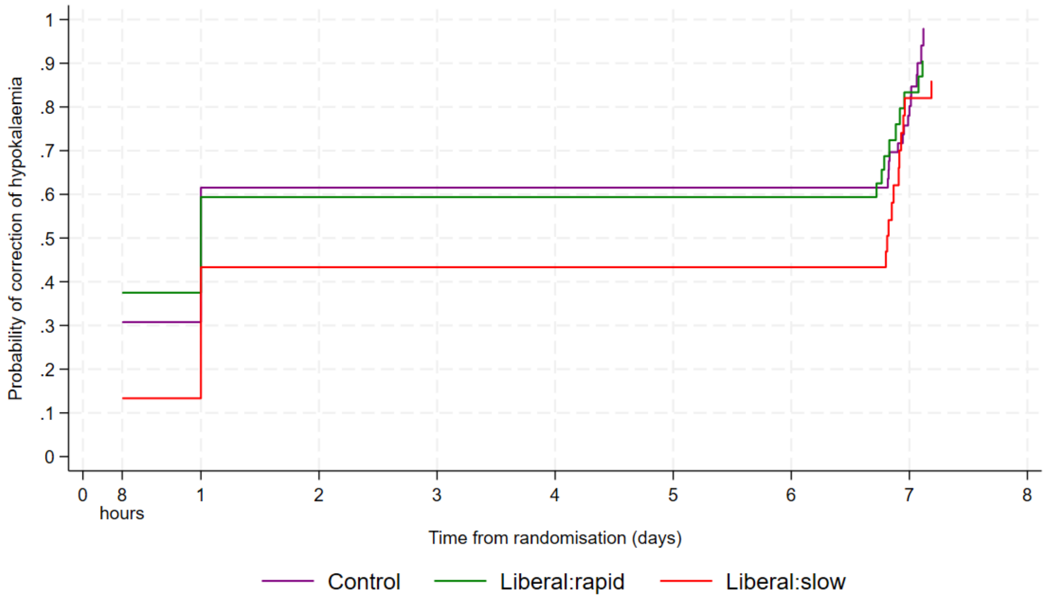

Figure S9: Residual Plots From All Linear Regression Models.

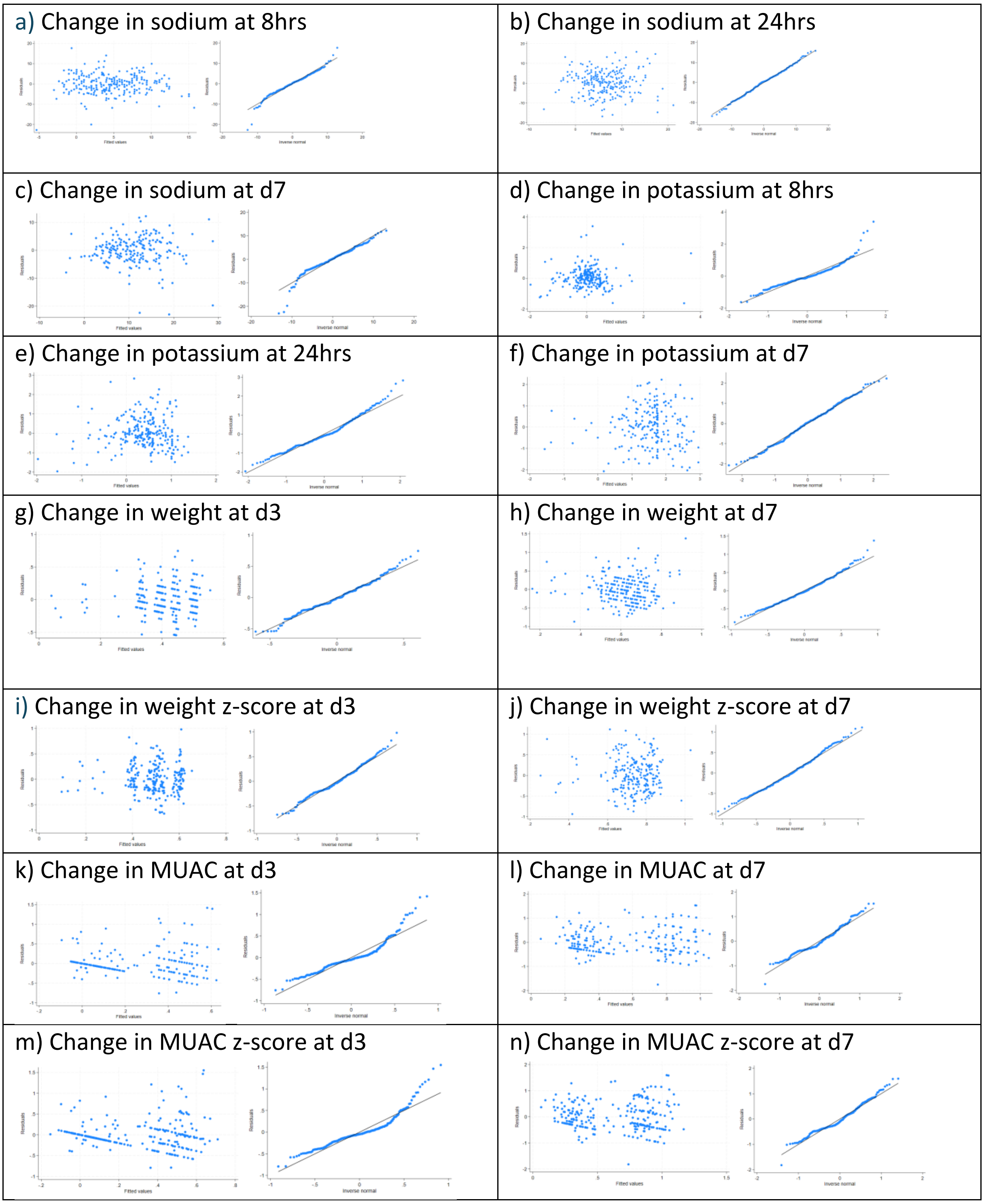

Table S1: Representativeness of Study Population

Category	Details
Disease, problem, or condition under investigation	Severe acute malnutrition (SAM) with severe dehydration due to gastroenteritis in children<br>*Globally, an estimated 19 million children under 5 years of age are affected by severe acute malnutrition (WHO fact sheet) with the highest rates occurring in sub-Saharan Africa and South-East Asia.*<br>*Amongst all pediatric admissions in sub-Saharan Africa (sSA) approximately 10%[1] of have severe malnutrition (SAM) and 49%-57% of SAM cases[2] are complicated by diarrhea.*
Sex and gender	Male children are more at risk of wasting[8,9], but sex differences are most likely caused by a complex interaction of social, environmental, physiological and genetic factors throughout the life cycle[10].<br>GastroSAM enrolled 54 % female, 46 % male distribution – across very different health-care levels and geographies, girls constitute 45–53 % of SAM-diarrhea admissions in Africa[2,3,11].
Age	Young age profile, reflecting epidemiology of SAM with diarrhea. The median age in GASTROSAM was 13 months (IQR 9–23), and 82% were <2 years.<br>This is in line with the known age distribution of SAM with diarrhea: Median age differs according to study enrollment criteria, e.g those enrolling children 6-23 months, median 11 months (IQR 7–16 months)[12], and those enrolling children 6 months-12 year, median 22 months [IQR 15 to 34][2]
Race or ethnic group	Participants wereAfrican children from diverse locations in Kenya, Uganda, Niger, and Nigeria. The sites in Nigeria and Niger which enrolled most participants cater to a diverse population consisting of multiple ethnic groups.
Geography	Multi-country trial across East Africa (Kenya, Uganda) and West Africa (Niger, Nigeria), capturing diverse malaria endemicity and healthcare settings. The sites also cover diverse climate zones and food security contexts (highly insecure in Niger/Nigeria and less so in Kenya/Uganda). The Nigeria site is also a humanitarian emergency context, rife with displacement. The urban and rural contexts are both captured as well.<br><br>The origins of malnutrition are complex with the interplay of several factors, access to or inadequate diet, underlying medical conditions, and socioeconomic circumstances. It is endemic through-out much of sSA in addition to seasonal epidemics due to the ''Hunger gap'' – referring to a period of acute food insecurity, typically between April and September, when populations are between harvests. In the Sahel (including Niger and

	northern Nigeria) this is a recurring problem leading to food shortages and a surge in pediatric malnutrition cases. It is also common amongst displaced populations experiencing conflict and humanitarian crises.<br>Most uncomplicated cases are managed in community feeding centers, whereas those with severe and complicated malnutrition (SAM) are hospitalized. Overall mortality across Africa for children with SAM is 18-20%[1,12].
Other considerations	Median age of all children in Africa hospitalized with all gastroenteritis is 13 months (IQR 8-21); 34% have severe dehydration and the overall in-hospital mortality is 4%. Risk factors for fatal outcome include HIV and severe malnutrition[13].<br>In Africa, observational studies indicate that 49-60% of SAM admission are complicated by diarrhea[1,3,14]; 30% of children with SAM with diarrhea have severe dehydration[2]; 8% have bacteremia; 12% have severe hyponatremia (sodium<125mmol/l) and 35% have severe hypokalemia [2] all risk factors for poor outcome. In studies reporting to follow international guidelines for rehydration (oral) mortality for children with diarrhea and severe dehydration ranges from 27%-41%[1-3].<br><br>Our trial population is children with severe acute malnutrition (wasting), with 4% participants with kwashiorkor. Globally, the prevalence of kwashiorkor is uncertain[15], with high geographical distribution and can range anywhere between 0-32%[16].<br>HIV low prevalence contexts, malaria rapid test- positive 17 %, also typical for the region.<br>Excluded congenital/rheumatic heart disease and chronic diarrhea >14 days (<1 % of admissions).<br>HIV low prevalence contexts, malaria rapid test- positive 17 %, also typical for the region.<br>In general, pediatric wards lack fully equipped ICU facilities, and critically ill children are typically managed limited resources (no mechanical ventilation, no invasive monitoring) which mirror usual care in African setting. In the GASTROSAM trial in Niger and Nigeria included a dedicated trial team who were responsible for all management throughout 24 hours. Children were managed on high dependency units and discharged when their nutritional status had improved. This meant a prolonged period of close clinical surveillance. For the children in Uganda there was no dedicated high dependency unit; so they were managed by a mixture of study and ward staff.
Overall representativeness	The Participants in the GASTROSAM trial are representative of children hospitalized with SAM and gastroenteritis in Africa. The participants were predominantly infants and young children (median ~1 year) with an equal

	sex distribution and a high burden of diarrheal illness – characteristics that match the profile of complicated SAM cases seen in African hospitals.<br>In terms of severity of the overall cohort the baseline characteristics of participants included a higher proportion of children who had risk factors had with previously identified for fatal outcome. These included impaired consciousness (39%); bacteremia (largely gram negative) (12%)); severe hyponatremia sodium < 125 mmol/L (52%); severe hypokalemia < 2.5 mmol/L (45%) and a previous admission with SAM (30%)[2]. Few had the kwashiorkor phenotype or HIV. However, the presence of either HIV or kwashiorkor are not major effect modifiers for children with SAM and severe dehydration due to diarrhea[2].

References

1. Maitland K, Berkley JA, Shebbe M, Peshu N, English M, Newton CR. Children with severe malnutrition: can those at highest risk of death be identified with the WHO protocol? PLoS medicine 2006;3(12):e500. (In eng)
2. Talbert A, Thuo N, Karisa J, et al. Diarrhoea complicating severe acute malnutrition in Kenyan children: a prospective descriptive study of risk factors and outcome. PLoS One 2012;7(6):e38321. (In eng). DOI: 10.1371/journal.pone.0038321.
3. Tamir TT, Zegeye AF, Workneh BS, et al. Childhood wasting and associated factors in Africa: evidence from standard demographic and health surveys from 35 countries. BMC Public Health 2025;25:454.
4. Thurstans S, Opondo C, Seal A, et al. Boys are more likely to be undernourished than girls: a systematic review and meta-analysis of sex differences in undernutrition. BMJ global health 2020;5(12):e004030.
5. Thurstans S, Opondo C, Seal A, et al. Understanding sex differences in childhood undernutrition: a narrative review. Nutrients 2022;14(5):948.
6. Chiabi A, Malangue B, Nguefack S, et al. The clinical spectrum of severe acute malnutrition in children in Cameroon: a hospital-based study in Yaounde, Cameroon. Translational pediatrics 2017;6(1):32.
7. Irena AH, Mwambazi M, Mulenga V. Diarrhea is a major killer of children with severe acute malnutrition admitted to inpatient set-up in Lusaka, Zambia. Nutrition journal 2011;10:1-6.
8. Berkley JA, Ngari M, Thitiri J, et al. Daily co-trimoxazole prophylaxis to prevent mortality in children with complicated severe acute malnutrition: a multicentre, double-blind, randomised placebo-controlled trial. The Lancet Global Health 2016;4(7):e464-e473.
9. Talbert A, Ngari M, Bauni E, et al. Mortality after inpatient treatment for diarrhea in children: a cohort study. BMC medicine 2019;17:1-11.
10. Berkley JA, Ngari M, Thitiri J, et al. Daily co-trimoxazole prophylaxis to prevent mortality in children with complicated severe acute malnutrition: a multicentre, double-blind, randomised placebo-controlled trial. Lancet Glob Health 2016;4(7):e464-73. DOI: 10.1016/S2214-109X(16)30096-1.
11. Bhutta ZA, Berkley JA, Bandsma RH, Kerac M, Trehan I, Briend A. Severe childhood malnutrition. Nature reviews Disease primers 2017;3(1):1-18.
12. Frison S, Checchi F, Kerac M. Omitting edema measurement: how much acute malnutrition are we missing? The American journal of clinical nutrition 2015;102(5):1176-1181.

Table S2: Intravenous Fluid Administration Rates for Liberal:rapid arm

No Shock and Aged >1 year	STEP 1: (30mls/kg over 30 mins)		STEP 2: (70mls/kg over 2.5 hours)	
**Weight**	**Volume**	**Drops/min**	**Volume**	**Drops/min**
2 kg	60	40	140	19
3 kg	90	60	210	28
4 kg	120	80	280	37
5 kg	150	100	350	47
6 kg	180	120	420	56
7 kg	210	140	490	65
8 kg	240	160	560	76
9 kg	270	180	630	84
10 kg	300	200	700	93
11 kg	330	220	770	103
12 kg	360	240	840	112
13 kg	390	260	910	121
14 kg	420	280	980	131
15 kg	450	300	1050	140
**No Shock and Aged >1 year**	STEP 1: (30mls/kg over 1 hour)		STEP 2: (70mls/kg over 5 hours)	
2 kg	60	20	140	9
3 kg	90	30	210	14
4 kg	120	40	280	19
5 kg	150	50	350	23
6 kg	180	60	420	28
7 kg	210	70	490	32
8 kg	240	80	560	37
9 kg	270	90	630	42
10 kg	300	100	700	47
11 kg	330	110	770	51
12 kg	360	120	840	56
13 kg	390	130	910	61
14 kg	420	140	980	65
15 kg	450	150	1050	70

Table S3: Intravenous Fluid Administration Rates for Liberal:slow arm

(100mls/kg over 8 hours)		
**Weight**	**Volume**	**Drops/min**
2 kg	200	8
3 kg	300	13
4 kg	400	17
5 kg	500	21
6 kg	600	25
7 kg	700	29
8 kg	800	33
9 kg	900	36
10 kg	1000	42
11 kg	1100	46
12 kg	1200	50
13 kg	1300	54
14 kg	1400	58
15 kg	1500	63

Table S4: Intravenous Fluid Administration Rates for Boluses Given to Those in Shock in Control Arm

(15mls/kg over 1 hour)		
**Weight**	**Volume**	**Drops/min**
2 kg	30	10
3 kg	45	15
4 kg	60	20
5 kg	75	25
6 kg	90	30
7 kg	105	35
8 kg	120	40
9 kg	135	45
10 kg	150	50
11 kg	165	55
12 kg	180	60
13 kg	195	65
14 kg	210	70
15 kg	225	75

Table S5: Details of Rehydration Solutions

**Ringers Lactate**	**D5 Ringers Lactate**<br>**Darrows Solution**	**Normal Saline**
Per 100ml	Per 100 ml	Per 100 ml
Sodium Chloride (Na Cl) (0.6g)	Sodium Chloride (0.2g)	Sodium Chloride (0.9g)
Potassium Chloride (K Cl) (0.04g)	Potassium Chloride (0.13g)	
Sodium Lactate 0.32g	Sodium Lactate 0.3g	
Concentration in mmol/L<br>Na 131, K 5, Cl 111<br>Bicarbonate (as lactate) 29	Concentration in mmol/L<br>N 61, K 17, Cl 52<br>Bicarbonate (lactate) 20	Concentration in mmol/L<br>Na 154

Table S6: Further Detail on Adherence to Intravenous Rehydration Strategy

	**Control**	**Liberal: rapid**		**Liberal:slow**	**Total**
	**All ages (N=138)**	**Under 1 yr old (N=27)**	**Over 1 yr old (N=40)**	**All ages (N=67)**	**All ages (N=272)**
Protocol strategy	In shock: 15mls/kg over 1 hour, repeated once if necessary. No further IV rehydration.	In shock: 20mls/kg as quickly as possible Not in shock: 30mls/kg over 1 hour. Then 70mls/kg over 5 hours.	In shock: 20mls/kg as quickly as possible Not in shock: 30mls/kg over 1 hour. Then 70mls/kg over 5 hours.	No additional treatment for shock. 100mls/kg over 8 hours.	
Total volume of bolus fluid received** (mls/kg)	15 (15-23)	20 (20-20)	20 (20-20)	-	20 (15-20)
Total duration of bolus fluid received** (minutes)	60 (60-120)	15 (15-15)	15 (15-15)	-	60 (15-120)
Total volume of rehydration fluid received by IV (mls/kg)	0 (0-0)	100 (100-100)	100 (100-100)	100 (100-100)	53 (0-100)
Total duration of rehydration fluid received by IV (hours)	0 (0-0)	6 (6-6)	3 (3-3)	8 (8-8)	2 (0-6)
Number receiving a blood transfusion within 24 hours of randomisation	4 (8%)	1 (4%)	0 (0%)	0 (0%)	5

**Of those that received initial bolus.
Note: Showing n (col %) or median IQR.

Table S7: Other Outcomes from Statistical Analysis Plan: Liberal vs. Control; Liberal:rapid vs. Control and Liberal:slow vs. Control.

	**Control (N=138)°**	**Liberal (rapid and slow) (N=134)°**	**Liberal:rapid (N=64)°**	**Liberal:slow (N=64)°**	**Liberal vs. control estimate (95% CI)**	**Liberal:rapid vs. control estimate (95% CI)**	**Liberal:slow vs. control estimate (95% CI)**
Change in weight-for-age z-score at 3 days	0.4 (0.3)	0.5 (0.3)	0.5 (0.3)	0.6 (0.4)	0.1 (0.0, 0.2)[a]	0.1 (0.0, 0.2)[a]	0.1 (0.0, 0.2)[a]
Change in MUAC-for-age z-score at 3 days	0.2 (0.4)	0.4 (0.5)	0.3 (0.5)	0.4 (0.4)	0.1 (0.0, 0.2)[a]	0.1 (0.0, 0.2)[a]	0.1 (0.0, 0.2)[a]
Change in weight-for-age z-score at 7 days	0.7 (0.4)	0.7 (0.4)	0.7 (0.4)	0.8 (0.4)	0.0 (-0.1, 0.1)[a]	0.0 (-0.1, 0.1)[a]	0.1 (0.0, 0.2)[a]
Change in MUAC-for-age z-score at 7 days	0.6 (0.6)	0.6 (0.6)	0.6 (0.7)	0.6 (0.6)	-0.0 (-0.1, 0.1)[a]	0.0 (-0.2, 0.2)[a]	0.0 (-0.2, 0.2)[a]
Severe hyponatremia at 7 days	6/109 (6%)	2/103 (2%)	2/50 (4%)	0/53 (0%)	0.33 (0.07, 1.74)[b]	0.71 (0.14, 3.70)[b]	NA[c]
Hypernatraemia at 7 days	2/109 (2%)	1/103 (1%)	0/50 (0%)	1/53 (2%)	0.52 (0.05, 5.91)[b]	NA[c]	1.02 (0.09, 11.70)[b]
Severe hypokalemia at 7 days	3/109 (3%)	6/103 (6%)	4/50 (8%)	2/53 (4%)	2.19 (0.53, 9.04)[b]	3.07 (0.65, 14.51)[b]	1.38 (0.22, 8.60)[b]
Change in sodium at 7 days	11.2 (7.6)	11.0 (7.8)	10.5 (8.2)	11.5 (7.5)	0.1 (-1.3, 1.5)[a]	-1.1 (-2.9, 0.8)[a]	1.3 (-0.4, 2.9)[a]
Change in potassium at 7 days	1.5 (1.1)	1.6 (1.3)	1.7 (1.3)	1.5 (1.2)	0.0 (-0.2, 0.3)[a]	0.1 (-0.3, 0.4)[a]	0.0 (-0.3, 0.3)[a]

°mean (sd) or n(%) presented [a]Difference in means (95% CI). [b]Odds ratio (95%CI) [c]Not enough events to calculate an estimate.

Note: The widths of confidence intervals for estimates have not been adjusted for multiplicity and should not be used in place of hypothesis testing.

Table S8: Primary, Secondary and Other Outcomes: Liberal:rapid vs. Control and Liberal:slow vs. Control Separately

	**Estimate (95% CI): Liberal:rapid intravenous rehydration vs. control**	**Estimate (95% CI): Liberal:slow vs. control**
**Primary outcome**		
**Mortality at 96 hours**	1.16 (0.40, 3.40)[a]	0.89 (0.28, 2.80)[a]
**Secondary outcomes**		
Mortality at 28 days	0.95 (0.40, 2.26)[b]	0.70 (0.27, 1.82)[b]
Urine output at 8 hours** (mls)	53 (-40, 146)[c]	128 (-46, 303)[c]
Time to correction of severe hyponatremia - hazard ratio	1.47 (0.99, 2.18)	1.72 (1.11, 2.67)
Time to correction of severe hypokalemia - hazard ratio	0.95 (0.61, 1.49)	0.75 (0.47, 1.21)
Severe hyponatremia at 8 hours	0.31 (0.15, 0.64)[d]	0.15 (0.06, 0.38)[d]
Severe hypokalemia at 8 hours	1.65 (0.88, 3.10)[d]	1.94 (1.04, 3.63)[d]
Change in sodium level at 24 hours from post-IV levels (8 hours)	-0.2 (-2.1, 1.7)[c]	0.4 (-1.5, 2.2)[c]
Change in weight (kg) at day 3	0.1 (0.1, 0.2)[c]	0.1 (0.0, 0.2)[c]
Change in MUAC (cm) at day 3	0.1 (-0.0, 0.2)[c]	0.1 (0.0, 0.2)[c]
Change in weight (kg) at day 7	0.0 (-0.1, 0.1)[c]	0.1 (-0.0, 0.2)[c]
Change in MUAC (cm) at day 7	0.0 (-0.2, 0.2)[c]	0.0 (-0.2, 0.1)[c]
**Other outcomes**		
Hypernatraemia at 8 hours	NA[e]	2.04 (0.28, 15.00)[d]
Change in sodium at 8 hours	5.4 (3.9, 7.0)[c]	6.1 (4.5, 7.6)[c]
Change in potassium at 8 hours	-0.3 (-0.5, -0.1)[c]	-0.3 (-0.5, -0.1)[c]
Severe hyponatremia at 24 hours	0.68 (0.33, 1.41)[d]	0.39 (0.17, 0.91)[d]
Hypernatraemia at 24 hours	2.07 (0.40, 10.61)[d]	2.10 (0.41, 10.80)[d]
Severe hypokalemia at 24 hours	1.48 (0.73, 3.01)[d]	1.69 (0.84, 3.39)[d]
Change in sodium at 24 hours	3.5 (1.6, 5.5)[c]	4.6 (2.8, 6.5)[c]
Change in potassium at 24 hours	-0.4 (-0.6, -0.1)[c]	-0.5 (-0.7, -0.2)[c]

[a]Risk ratio (95%CI), Mantel-Haenszel adjustment for site. [b]Hazard ratio (95%CI) (Cox regression) [c]Difference in means (95% CI) [d]Odds ratio (95%CI) † subhazard ratio, with death as a competing risk. **primary endpoint in previous protocol versions. Not actively collected at MSF sites. [e]Not enough events to calculate an estimate. Severe hyponatremia defined as sodium <125 mmol/L. Severe hypokalemia defined as potassium <2.5 mmol/L.

Note: The widths of confidence intervals for estimates have not been adjusted for multiplicity and should not be used in place of hypothesis testing.

Table S9: Subgroup Analysis for Primary Endpoint of Mortality at 96 Hours

**Subgroup**		**Control N (%)**	**Liberal (rapid and slow) N (%)**	**Liberal vs. control: Risk ratio (95% CI)**	**Interaction effect (95% CI)**
**Pre-specified subgroup analyses**					
ORS randomisation	Resomal	6/69 (9%)	5/68 (7%)	1.07 (0.36, 3.19)	1.00
	WHO ORS	5/69 (7%)	4/66 (6%)	0.98 (0.27, 3.54)	0.91 (0.18, 4.63)
Age	<1	1/51 (2%)	4/56 (7%)	3.45 (0.43, 27.91)	1.00
	≥1	10/87 (11%)	5/78 (6%)	0.72 (0.25, 2.07)	0.21 (0.02, 2.32)
Consciousness level	A	1/88 (1%)	3/80 (4%)	3.31 (0.34, 31.88)	1.00
	V	2/35 (6%)	2/43 (5%)	0.76 (0.10, 5.57)	0.23 (0.01, 4.91)
	P/U	8/15 (53%)	4/11 (36%)	0.90 (0.32, 2.56)	0.27 (0.02, 3.61)
Respiratory distress	No	4/115 (3%)	7/114 (6%)	1.75 (0.55, 5.53)	1.00
	Yes	7/23 (30%)	1/19 (5%)	0.31 (0.04, 2.48)	0.18 (0.02, 1.93)
**Post-hoc subgroup analyses**					
Country	Uganda	1/4 (25%)	1/5 (20%)	0.80 (0.07, 9.22)	1.00
	Nigeria	2/57 (4%)	4/56 (7%)	2.04 (0.39, 10.71)	2.54 (0.13, 48.87)
	Niger	3/70 (4%)	4/69 (6%)	1.35 (0.31, 5.84)	1.69 (0.10, 29.20)
	Kenya	5/7 (71%)	0/4 (0%)	Not estimable	Not estimable

Table S10: Safety Events

	Control (N=138)	Liberal:rapid (N=67)	Liberal:slow (N=67)	Total
Ever had an SAE	32 (23%)	14 (21%)	10 (15%)	56 (21%)
Number of SAEs	33	14	10	57
**SAE Criteria (% of SAEs):**				
Fatal	17 (52%)	8 (57%)	6 (60%)	31 (54%)
Life-threatening	12 (36%)	3 (21%)	1 (10%)	16 (28%)
Hospitalisation	3 (9%)	2 (14%)	3 (30%)	8 (14%)
Resulting in disability or incapacity	0 (0%)	0 (0%)	0 (0%)	0 (0%)
Other	1 (3%)	1 (7%)	0 (0%)	2 (4%)
**Relationship to IV fluids (% of SAEs):**				
Definitely	0 (0%)	0 (0%)	0 (0%)	0 (0%)
Probably	0 (0%)	0 (0%)	0 (0%)	0 (0%)
Possibly	0 (0%)	1 (7%)[3]	1 (10%)[2]	2 (4%)
Unlikely	18 (55%)	7 (50%)	3 (30%)	28 (49%)
Unrelated	14 (42%)	6 (43%)	6 (60%)	26 (46%)
Not assessed	1 (3%)	0 (0%)	0 (0%)	1 (2%)

[2] Death: severe malnutrition, gastroenteritis and initial shock. The child was referred at Day 7 to a specialist teaching hospital as their nutritional condition was not improving. The parents later self-discharged and reported that the child died at home.
[3] Readmission to hospital 13 days after randomisation but no signs of heart failure.

Table S11: List of SAEs

SAEs occurring up to 28 days after randomisation are presented below.

	SAE criteria	Event description
1	Death	cardiopulmonary arrest
2	Death	cardiopulmonary arrest
3	Death	cardiopulmonary arrest
4	Death	death
5	Death	death
6	Death	death
7	Death	death
8	Death	death
9	Death	death + severe malnutrition + gastroenteritis + shock
10	Death	gastroenteritis + malaria + pneumonia
11	Death	gastroenteritis + malaria + pneumonia + septic shock
12	Death	gastroenteritis + malnutrition
13	Death	gastroenteritis + severe malaria + electrolyte imbalance + sepsis
14	Death	malaria + death + shock
15	Death	pneumonia + sepsis + skin lesion
16	Death	probable peritonitis
17	Death	sepsis
18	Death	sepsis
19	Death	sepsis
20	Death	sepsis + death
21	Death	sepsis + death
22	Death	sepsis + severe malaria + malnutrition
23	Death	sepsis + shock + death
24	Death	severe malnutrition + gastroenteritis + otitis media + sepsis
25	Death	severe pneumonia
26	Death	severe pneumonia + severe malaria + gastroenteritis + malnutrition
27	Death	shock + severe malnutrition + gastroenteritis
28	Death	shock + severe pneumonia + sepsis + hypoglycaemia
29	Death	suspected neurological event
30	Death	unknown
31	Death	worsening sepsis
32	Life threatening	circulatory impairment
33	Life threatening	circulatory impairment
34	Life threatening	gastroenteritis + shock
35	Life threatening	gastroenteritis + shock
36	Life threatening	gastroenteritis + shock
37	Life threatening	gastroenteritis + shock
38	Life threatening	gastroenteritis + shock + sepsis
39	Life threatening	hypovolemic shock
40	Life threatening	hypovolemic shock
41	Life threatening	hypovolemic shock + gastroenteritis + malaria
42	Life threatening	hypovolemic shock + malaria + sepsis + skin ulcers
43	Life threatening	hypovolemic shock + pneumonia + malaria

	SAE criteria	Event description
44	Life threatening	hypovolemic shock + sepsis
45	Life threatening	probable septic shock + sepsis
46	Life threatening	profuse diarrhea + sepsis
47	Life threatening	shock
48	Caused or prolonged hospitalisation	gastroenteritis + kwashiorkor
49	Caused or prolonged hospitalisation	gastroenteritis + lower respiratory infection
50	Caused or prolonged hospitalisation	gastroenteritis + pneumonia
51	Caused or prolonged hospitalisation	hypovolemic shock + sepsis + malaria + pneumonia
52	Caused or prolonged hospitalisation	pneumonia + anorexia + oral and genital candidiasis + malaria
53	Caused or prolonged hospitalisation	pneumonia + malaria + gastroenteritis
54	Caused or prolonged hospitalisation	readmission
55	Caused or prolonged hospitalisation	severe anaemia
56	Other	gastroenteritis + readmission + anemia
57	Other	pneumonia + anorexia

Table S12: Secondary Outcomes for the Liberal vs. Control Comparison Estimated with Multiple Imputation

	**Liberal vs. control: Estimate of association (95% CI) (complete case)**	**Liberal vs. control: Estimate of association (95%CI) (multiple imputation)**
**Secondary outcomes**		
Severe hyponatremia at 8 h	0.23 (0.12, 0.41) [d]	0.23 (0.13, 0.41) [d]
Severe hypokalemia at 8 h	1.79 (1.07, 2.99) [d]	1.82 (1.09, 3.04) [d]
Change in sodium level at 24 hours from post-IV levels (8 hours)	0.1 (-1.4, 1.6)[c]	0.3 (-1.2, 1.9) [c]
Weight change (kg) at Day 3	0.1 (0.1, 0.2)[c]	0.1 (0.0, 0.2) [c]
MUAC change (cm) at Day 3	0.1 (0.0, 0.2)[c]	0.1 (0.0, 0.2) [c]
Weight change (kg) at Day 7	0.0 (-0.0, 0.1)[c]	0.0 (-0.0, 0.1) [c]
MUAC change (cm) at Day 7	0.0 (-0.1, 0.1)[c]	-0.0 (-0.1, 0.1) [c]
**Other outcomes**		
Change in sodium at 8 h (mmol/L)	5.7 (4.5, 7.0)[c]	5.8 (4.6, 7.1) [c]
Hypernatremia at 8 h	1.01 (0.14, 7.27) [d]	1.13 (0.16, 8.08) [d]
Change in potassium (mmol/L) at 8 h	-0.3 (-0.5, -0.2)[c]	-0.4 (-0.5, -0.2) [c]
Change in sodium at 24 h	4.1 (2.6, 5.7)[c]	4.1 (2.6, 5.7) [c]
Severe hyponatremia at 24 h	0.53 (0.29, 0.98)[d]	0.51 (0.28, 0.94) [d]
Hypernatremia at 24 hours	2.08 (0.51, 8.51) [d]	2.15 (0.53, 8.78) [d]
Severe hypokalemia at 24 h	1.58 (0.89, 2.84) [d]	1.56 (0.88, 2.78) [d]
Change in potassium at 24 h	-0.4 (-0.6, -0.2)[c]	-0.4 (-0.6, -0.2) [c]

[c]Difference in means (95% CI)
[d]Odds ratio (95%CI)
Note: The widths of confidence intervals for estimates have not been adjusted for multiplicity and should not be used in place of hypothesis testing.

REFERENCES

1. Maitland K, Berkley JA, Shebbe M, Peshu N, English M, Newton CR. Children with severe malnutrition: can those at highest risk of death be identified with the WHO protocol? PLoS medicine 2006;3(12):e500. (In eng) (http://www.ncbi.nlm.nih.gov/entrez/query.fcgi?cmd=Retrieve&db=PubMed&dopt=Citation&list_uids=17194194).
2. Talbert A, Thuo N, Karisa J, et al. Diarrhoea complicating severe acute malnutrition in Kenyan children: a prospective descriptive study of risk factors and outcome. PLoS One 2012;7(6):e38321. (In eng). DOI: 10.1371/journal.pone.0038321.
3. Irena AH, Mwambazi M, Mulenga V. Diarrhea is a major killer of children with severe acute malnutrition admitted to inpatient set-up in Lusaka, Zambia. Nutrition journal 2011;10:1-6.
4. Pocket book of hospital care for children 2nd edition. Guidelines for the management of common childhood illnesses. Geneva: World Health Oganization, 2013.
5. Maitland K, Kiguli S, Opoka RO, et al. Mortality after fluid bolus in African children with severe infection. N Engl J Med 2011;364(26):2483-95. DOI: 10.1056/NEJMoa1101549.
6. Akech SO, Karisa J, Nakamya P, Boga M, Maitland K. Phase II trial of isotonic fluid resuscitation in Kenyan children with severe malnutrition and hypovolaemia. BMC Pediatr 2010;10:71. (In eng). DOI: 1471-2431-10-71.
7. Obonyo N, Brent B, Olupot-Olupot P, et al. Myocardial and haemodynamic responses to two fluid regimens in African children with severe malnutrition and hypovolaemic shock (AFRIM study). Crit Care 2017;21(1):103. DOI: 10.1186/s13054-017-1679-0.
8. Tamir TT, Zegeye AF, Workneh BS, et al. Childhood wasting and associated factors in Africa: evidence from standard demographic and health surveys from 35 countries. BMC Public Health 2025;25:454.
9. Thurstans S, Opondo C, Seal A, et al. Boys are more likely to be undernourished than girls: a systematic review and meta-analysis of sex differences in undernutrition. BMJ global health 2020;5(12):e004030.
10. Thurstans S, Opondo C, Seal A, et al. Understanding sex differences in childhood undernutrition: a narrative review. Nutrients 2022;14(5):948.
11. Chiabi A, Malangue B, Nguefack S, et al. The clinical spectrum of severe acute malnutrition in children in Cameroon: a hospital-based study in Yaounde, Cameroon. Translational pediatrics 2017;6(1):32.
12. Berkley JA, Ngari M, Thitiri J, et al. Daily co-trimoxazole prophylaxis to prevent mortality in children with complicated severe acute malnutrition: a multicentre, double-blind, randomised placebo-controlled trial. The Lancet Global Health 2016;4(7):e464-e473.
13. Talbert A, Ngari M, Bauni E, et al. Mortality after inpatient treatment for diarrhea in children: a cohort study. BMC medicine 2019;17:1-11.
14. Berkley JA, Ngari M, Thitiri J, et al. Daily co-trimoxazole prophylaxis to prevent mortality in children with complicated severe acute malnutrition: a multicentre, double-blind, randomised placebo-controlled trial. Lancet Glob Health 2016;4(7):e464-73. DOI: 10.1016/S2214-109X(16)30096-1.
15. Bhutta ZA, Berkley JA, Bandsma RH, Kerac M, Trehan I, Briend A. Severe childhood malnutrition. Nature reviews Disease primers 2017;3(1):1-18.
16. Frison S, Checchi F, Kerac M. Omitting edema measurement: how much acute malnutrition are we missing? The American journal of clinical nutrition 2015;102(5):1176-1181.

5\. Maitland, K. et al. Children with Severe Malnutrition: Can Those at Highest Risk of Death Be Identified with the WHO Protocol? PLOS Med. 3, e500 (2006).
